# lncRNA NKILA Promotes Warburg Effect and Immune Escape in Intrahepatic Cholangiocarcinoma by Regulating the MTX1/TOMM40 Axis

**DOI:** 10.1155/mi/7712817

**Published:** 2025-12-22

**Authors:** Meiying Zhu, Hui Zhu, Zunqiang Zhou, Haiming Zheng, Zhixia Dong, Wenhong Dong, Xinjian Wan

**Affiliations:** ^1^ Digestive Endoscopic Center, Shanghai Sixth People’s Hospital Affiliated to Shanghai Jiao Tong University School of Medicine, Shanghai, 200233, China, sjtu.edu.cn; ^2^ Institute of Diagnostic and Interventional Radiology, Shanghai Sixth People’s Hospital Affiliated to Shanghai Jiao Tong University School of Medicine, Shanghai, 200233, China, sjtu.edu.cn; ^3^ Department of Surgery, Shanghai Sixth People’s Hospital Affiliated to Shanghai Jiao Tong University School of Medicine, Shanghai, 200233, China, sjtu.edu.cn

**Keywords:** glycolytic reprogramming, immune escape, intrahepatic cholangiocarcinoma, metaxin 1/translocase of outer mitochondrial membrane 40, nuclear transcription factor NF-κB interacting lncRNA

## Abstract

**Background and Aims:**

Intrahepatic cholangiocarcinoma (ICC) is an aggressive malignancy with high heterogeneity and poor prognosis. Long noncoding RNAs (lncRNAs) play critical roles in tumorigenesis through dysregulated expression. We explored the effects and mechanisms of nuclear transcription factor NF‐κB interacting lncRNA (NKILA) in ICC.

**Methods:**

Bioinformatic analysis was performed to determine the expression and relationship of NKILA with metaxin 1 (MTX1), and translocase of outer mitochondrial membrane 40 (TOMM40) expression in ICC tissue samples. Cholangiocarcinoma cell lines were cultured in vitro and the transplanted tumor model was constructed in vivo to study the role of NKILA in ICC. Immunohistochemistry (IHC), Western blot, and quantitative real‐time polymerase chain reaction (qRT‐PCR) were used to detect the effects of NKILA on the Warburg effect, autophagy, programed cell death 1 ligand 1 (PD‐L1) expression, and CD8^+^ T cytotoxicity in ICC cells. RNA immunoprecipitation (IP) (RIP) assay and RNA–RNA pull down assays were utilized to detect the binding of NKILA and MTX1, and CO‐IP was performed to assess the interaction between MTX1 and TOMM40.

**Results:**

We found that NKILA, MTX1, and TOMM40 were substantially upregulated in ICC tissues, and NKILA silencing reduced MTX1‐TOMM40 binding in ICC cells. NKILA facilitated proliferation, invasion, Warburg effects, and autophagy of ICC cells by regulating mammalian target of rapamycin (mTOR) pathway, PD‐L1 expression, and CD8^+^ T cytotoxicity, while dichloroacetate (DCA) could reverse these effects. Mechanistically, NKILA binds directly to MTX1 mRNA, which stabilizes MTX1 mRNA and thereby promotes the expression of MTX1 protein. NKILA silencing could inactivate MTX1/TOMM40 axis to inhibit Warburg effect and autophagy‐associated immune escape.

**Conclusions:**

LncRNA NKILA promotes Warburg effect and immune escape in ICC by regulating the MTX1/TOMM40 axis.

## 1. Introduction

Intrahepatic cholangiocarcinoma (ICC) is the second most common primary liver cancer following hepatocellular carcinoma, characterized by high metastasis rate, high chemotherapy resistance, and hidden onset. Consequently, most patients present with local invasion or distant metastasis at diagnosis, precluding curative radical surgical treatment [1]. The long‐term use of first‐line chemotherapy regimens for advanced patients is prone to drug resistance and adverse drug reactions [2]. During the development and progression of ICC, abnormal endogenous small molecule metabolites such as sugars, amino acids, and lipids have been detected in the patient’s organism [3, 4]. Tumor cells exhibit unique metabolic and bioenergetic profiles [5]. Metabolic plasticity enables cancer cells to adapt to a suboptimal environment and optimizes cell development by sustaining survival, proliferation, and metastasis [3, 5]. Targeting this tumor cell‐specific energy metabolism represents a promising therapeutic route.

Our previous study demonstrated the nuclear transcription factor NF‐κB interacting long noncoding RNA (lncRNA) (NKILA) as a regulator of cholangiocarcinoma cell growth [6]. LncRNAs can affect various cellular functions through mediating transcriptional and post‐transcriptional processes, and also regulate the immune escape or immune resistance of tumors [7, 8]. However, the exact mechanism of NKILA on ICC metabolism remains unclear. According to previous findings that NKILA is mostly localized in the cytoplasm and partially in the nucleus [9], we speculated NKILA may affect the transcription of certain RNAs to regulate the progression of ICC, in addition to competitively binding miRNAs and preventing RelA (p65) from entering the nucleus.

ENCORI analysis identified four NKILA‐binding RNAs, alpha‐1,3‐glucosyltransferase (ALG6), sushi domain containing 6 (SUSD6), actin filament associated protein 1 (AFAP1), and metaxin 1 (MTX1), which were differentially expressed in cholangiocarcinoma tissues compared to adjacent tissues in GEPIA analysis. Of them, MTX1, a protein located in mitochondria (GeneCard website), may be involved in the Warburg process, but has not been sufficiently demonstrated [10]. Currently, comprehensive investigations have elucidated the significance of the Warburg effect in cancer development [11–13]. Search tool for recurring instances of neighboring genes (STRING) analysis revealed that MTX1 could interact with translocase of outer mitochondrial membrane 40 (TOMM40) that participates in the maintenance of mitochondrial function [14, 15]. Furthermore, GEPIA analysis unveiled upregulation of TOMM40 in cholangiocarcinoma. Accordingly, we hypothesized that NKILA may promote Warburg effect in cancers via upregulating MTX1 and enhancing its interaction with TOMM40.

Autophagy can facilitate tumorigenesis, especially in ICC. It is well known that mammalian target of rapamycin (mTOR) is a classical autophagy‐related pathway, and its activation can inhibit autophagy [16]. Intriguingly, aerobic glycolysis regulates autophagy, and Warburg‐like metabolism can activate the mTOR pathway [17]; for instance, the Warburg‐associated protein pyruvate kinase (PK) isoenzyme type 2 (PKM2) can stimulate the mTOR pathway [18, 19]. Studies have shown that the autophagy pathway can reduce the expression of programmed cell death 1 ligand 1 (PD‐L1) [20]. Therefore, based on the important role of NKILA, Warburg effect, and autophagy in the development of cholangiocarcinoma, we conjectured that NKILA enhances the Warburg effect, suppresses autophagy and PD‐L1 degradation, and promotes immune escape of ICC by elevating MTX1 and TOMM40 expressions, and meanwhile collected clinical, in vivo, and in vitro evidences.

## 2. Methods

### 2.1. Bioinformatics Analysis

The University of ALabama at Birmingham CANcer data analysis portal (UALCAN) (https://ualcan.path.uab.edu/) was used to analyze the expressions of NKILA, MTX1, and TOMM40 in cholangiocarcinoma. ENCORI (https://starbase.sysu.edu.cn/) was employed for analyzing the binding site of NKILA to MTX1. STRING (https://string-db.org/) was exploited to dissect the interacting proteins of MTX1.

### 2.2. Ethics Statement

Clinical or animal assays were approved by Ethics Committees of Shanghai Sixth People’s Hospital (2022‐YS‐285) or Zhejiang Baiyue Biotech Co., Ltd for Experimental Animals Welfare (ZJBYLA‐IACUC‐20231018). Clinical samples contained tumor tissues and adjacent tissues (nonmalignant bile duct tissue) from 10 patients with ICC. Animal experiments were conducted in accordance with the guidelines for the management and use of experimental animals.

### 2.3. Immunohistochemistry (IHC)

Tumor tissues or adjacent tissues were dehydrated, embedded, and sliced into 4 μm sections. Then, the sections were baked at 70°C for 40 min, dewaxed, and rinsed three times (3 min/time) in phosphate buffered saline (PBS) buffer (pH 7.4) (P1020, Solarbio, China). After removal of endogenous peroxides (P0100A, Beyotime, China), the sections were repaired with sodium citrate antigen repair solution (P0081, Beyotime, China) for 20 min, incubated with 5% goat serum blocking solution (C0265, Beyotime, China) for 30 min, and cultured with 50 μL primary antibodies at 4°C overnight, glucose transporter type 1 (Glut1) (ab115730, 1/250, Abcam, UK), lactate dehydrogenase (LDH) (#3582, 1/200, cell signaling technology, USA), PKM2 (#4053, 1/400, cell signaling technology, USA) and light chain 3A/B (LC3A/B) (66139‐1‐IG, Proteintech, China). The next day, the sections were rewarmed at room temperature for 30 min, and the secondary antibody (ab6721 or ab205719, 1/1000, Abcam, UK) was added dropwise for 2 h incubation at room temperature. DAB (P0202, Beyotime, China) was used for color development. After hematoxylin restaining (C0107, Beyotime, China), gradient alcohol dehydration (A507050, Sangon Biotech, China), xylene transparentization (C04305302, Nanjing Reagent, China), and neutral dendrimer sealing (G8590, Solarbio, China), the positive cells were visualized under the microscope (Ti2, Nikon, Japan) (200×).

### 2.4. Quantitative Real‐Time Polymerase Chain Reaction (qRT‐PCR)

Utilizing Trizol reagent (15596026, Invitrogen, USA), total RNA was extracted from tissues or cells. The cDNA was synthesized with a reverse transcription kit (4368813, Applied Biosystems, USA), and then added with specific primers NKILA (forward primer: 5′‐GGCACTGACCGTCTTCTGTT‐3′, reverse primer: 5′‐GGGGTAGACGCTGGGTATTG‐3′), MTX1 (forward primer: 5′‐TGCTGACCTATGCCAGATTTACT‐3′, reverse primer: 5′‐TGTGTGGAACTGAGATGACCT‐3′), TOMM40 (forward primer: 5′‐AAGCTCACAGTCAACAAAGGG‐3′, reverse primer: 5′‐GGTAGTTGGACTCCCCGATTG‐3′), and β‐actin (forward primer: 5′‐CATGTACGTTGCTATCCAGGC‐3′, reverse primer: 5′‐CTCCTTAATGTCACGCACGAT‐3′). The PCR amplification system was formulated according to the instructions of SYBR GreenER qPCR SuperMix (1,176,202 K, Invitrogen, USA), with a total of 50 μL reactant. Amplification was performed using a preset reaction program (QuantStudio 3, A28137, Applied Biosystems), and the samples were collected at the end of the cycle for fluorescence quantitative PCR. The primer sequence was designed by Sangon Biotech (Shanghai) Co., Ltd.

### 2.5. Cells and Treatment

Human intrahepatic bile duct epithelial cells HIBEpic (YB‐H0110) were bought from Ybscience (China). ICC cells RBE (AW‐CELLS‐H0303) and HCCC‐9810 (AW‐CELLS‐H0107) were purchased from Shanghai Anwei Biotechnology Co., Ltd. (China), while HuCCT1 (CL‐0725) was ordered from Procell (China). The cancer cells were identified by short tandem repeat. Cells were cultured in Roswell Park Memorial Institute (RPMI) 1640 medium (12633012, ThermoFisher, USA) containing 10% fetal bovine serum (FBS) (11875093, 10099158, Gibco, USA) at 37°C and 95% humidity. HIBEpic cells were cultured with DMEM under the same conditions as cancer cells. About 20 mM dichloroacetate (DCA) (GC16795, GlpBio Technology, China), a Warburg effect inhibitor, was used to pretreat HuCCT1 cells (12 h) for in vitro experiments [21]. NKILA knockdown lentiviral vector (knockdown‐NKILA [KD‐NKILA], 5′‐CAGAAACTCTCCAAATATTAA‐3′), MTX1 overexpression lentiviral vector (overexpression‐MTX1 [OE‐MTX1]), TOMM40 knockdown plasmid (knockdown‐TOMM40 [KD‐TOMM40], 5′‐CAAAGGGUUGAGUAACCAUUUTT‐3′), and NKILA overexpression plasmid (overexpression‐NKILA, OE‐NKILA) were purchased from GenePharma (China). Lipofectamine 3000 reagent (L3000001, Invitrogen, USA) was utilized for cell transfection. The transfection efficiency was detected by qRT‐PCR.

### 2.6. 5‐Ethynyl‐2′‐ Deoxyuridine (EdU) Staining

BeyoClick EdU Cell Proliferation Kit with Alexa Fluor 647 (C0081S) was procured from Beyotime (China). HuCCT1 cells were incubated in 6‐well plates overnight, followed by the addition of 2 × of EdU working solution prewarmed at 37°C in equal volume for 2 h incubation. After EdU labeling of the cells, cells were fixed with 1 mL fixative (P0098, Beyotime, China) for 15 min at room temperature, incubated with 1 mL permeabilization solution (P0097, Beyotime, China) for 10–15 min at room temperature, and reacted with 0.5 mL Click reaction solution for 30 min at room temperature with gentle shaking in the dark. Hoechst 33,342 (C1029, Beyotime, China) was used for nuclei staining, followed by counting of EdU‐positive cells under the microscope (DMi8, Leica, Germany) (200×).

### 2.7. Terminal Dexynucleotidyl Transferase (TdT)‐Mediated dUTP Nick End Labeling (TUNEL) Assay

HuCCT1 cells in each treatment group were washed with PBS, fixed with 4% paraformaldehyde (P0099, Beyotime, China) for 30 min, and incubated with PBS resuspension cells containing 0.3% Triton X‐100 (P0096, Beyotime, China) at room temperature for 5 min. TUNEL detection solution (C1090, Beyotime, China) was added and repeatedly blown until mixed. Cells were incubated at 37°C for 1 h and washed with PBS. After smear treatment, five visual fields were randomly selected under fluorescence microscope (Ni‐U, NIKON, Japan) for observation (200×), where the total number of cells, the number of TUNEL‐positive cells, and the positive rate of TUNEL were calculated.

### 2.8. Western Blot

RIPA lysis buffer (89901, Thermo Scientific, USA) was added into HuCCT1 cells or tumor tissues for extracting total proteins, which were quantified with BCA Protein Assay Kit (BCA1, Sigma–Aldrich, USA). Proteins were boiled for 5 min after addition of protein sample buffer, then separated by sodium dodecyl sulfate‐polyacrylamide gel electrophoresis (SDS‐PAGE) (P0012A, Beyotime, China) and transferred to polyvinylidene difluoride membranes (IPVH08100, Millipore, USA). The membranes were blocked with 5% skimmed milk powders (1.15363, Millipore, USA), and probed with primary antibodies at 4°C overnight (N‐cadherin, ab18203, 140 kDa, 1 µg/mL, Abcam, UK; E‐cadherin, ZRB1692‐25 μL, 120 kDa, 1/10,000, Sigma, Germany; vimentin, ab137321, 56 kDa, 1/1000, Abcam, UK; Glut1, ab115730, 46 kDa, 1/100,000, Abcam, UK; LDH, #3582, 32 kDa, 1/1000, CST, USA; PKM2, #4053, 55 kDa, 1/1000, CST, USA; hexokinase2 (HK2), ab209847, 102 kDa, 1/1000, Abcam, UK; monocarboxylate transporter 1 (MCT1), #76508, 44 kDa, 1/1000, CST, USA; sequestosome 1 (P62), #39749, 62 kDa, 1/1000, CST, USA; Beclin 1, #3495, 60 kDa, 1/1000, CST, USA; LC3, #2775, 14 kDa, 16 kDa, 1/1000, CST, USA; phospho (p)‐mTOR, #5536, 289 kDa, 1/1000, CST, USA; mTOR, #2983, 289 kDa, 1/1000, CST, USA; PD‐L1, ab205921, 46 kDa, 1/1000, Abcam, UK; MXT1, 20139‐1‐AP, 32 kDa, 1/1000, Proteintech, China; TOMM40, ab185543, 38 kDa, 1/1000, Abcam, UK; β‐actin, ab8226, 44 kDa, 1 µg/mL, Abcam, UK). Then, the membranes were incubated with secondary antibodies (ab6702, 1/1000, Abcam, UK; ab6708, 1/3000, Abcam, UK) for 2 h. The target proteins were visualized by an ECL kit (CW0049M, CWBIOTECH, China).

### 2.9. Measurement of Warburg‐Associated Indicators

Glucose Uptake Assay Kit (ab136956), Pyruvate Assay Kit (ab65342), Lactate Colorimetric Assay Kit (ab282923), adenosine triphosphate levels (ATP) Colorimetric Assay Kit (ab83355), PK Activity Colorimetric Assay Kit (ab83432), and LDH A Activity assay kit (ab102526) were obtained from Abcam (UK). Briefly, cells were collected, washed with PBS, suspended in the corresponding assay buffer, and centrifuged at high speed for 2 min. The supernatant was incubated for 30 min with glucose/lactate/pyruvate/ATP/PK/LDHA reactants. Absorbance was measured at 412, 450, 570, or 590 nm using a microplate reader (VLBLATGD2, Thermo Fisher, USA).

### 2.10. Cytotoxicity Assay

CyQUANT LDH Cytotoxicity Assay Kit (V23111) was purchased from Invitrogen (USA). CD8^+^ T (effector) cells were cocultured with HuCCT1 (target) cells in serum‐free medium at a ratio of 5:1 for 24 h. About 50 μL medium was mixed with an equal volume of the reactants. The reaction was terminated with the stop solution, and a microplate reader was used to read the plate at A490 and A680 to analyze LDH activity.

### 2.11. Ribonuclease Protection Assay (RPA)

Each RNA sample from HuCCT1 cells was incubated for 1 h at 37°C and then treated with RNAse A/T mixture (EN0551, Thermo Scientific, USA) to digest the single‐stranded RNA. After incubation for 30 min at 37°C, the samples were treated with proteinase K (25530049, Invitrogen, USA). Following RPA, NKILA was detected using RT‐PCR and gel electrophoresis targeting the overlapping region of NKILA positive and antisense transcripts and the nonoverlapping region of NKILA, respectively.

### 2.12. RNA Immunoprecipitation (IP) (RIP) and PCR Detection

The binding ability of MTX1 to NKILA was detected using Magna RIP RNA‐Binding Protein IP Kit (17–700, Sigma–Aldrich, USA) according to the instructions. The cells are digested and collected using trypsin. Protease inhibitors and RNase inhibitors were added to the cells, and the cells were blown and mixed, and then cleaved on ice. The supernatant was centrifuged and placed in a centrifuge tube. Part of the supernatant was retained as the input group, and the rest was added to protein A + G beads and incubated at 4°C for 10 min. After magnetic separation, the supernatant was retained. The magnetic beads combined with DDX3X antibody (11115‐1‐AP, proteintech, USA) were added with 350 μL lysate and 400 μL cell lysate supernatant and were rotated at 4°C at 10 rpm/min for 2 h. When reaction is complete, place on magnetic rack and discard supernatant. The corresponding washing liquid was added to the magnetic bead compound, washed in vortex for 2 min, placed on the magnetic rack, discard the supernatant, and repeated 3–4 times. About 300 μL eluent was added to the magnetic bead complex. The eluent was swirled for 30 s and placed on the magnetic rack to collect the supernatant. The purified RNA was analyzed by PCR.

PCR Kit with Taq (D732, Beyotime, China) was used in part of PCR experiments. The IP RNA was eluted and purified by reverse transcription reaction. Appropriate amount of RNA was taken, reverse transcription primers, reverse transcriptase and other reagents were added, and cDNA was obtained by reverse transcription. The cDNA was then used as a template for PCR amplification. After amplification, PCR products were separated by electrophoresis on agarose gel stained with GelRed, and DNA bands were visualized under UV light.

### 2.13. RNA–RNA Pull down Assay

Biotin‐labeled full‐length sense NKILA and antisense transcripts (or its truncated/mutant fragments) was in vitro transcribed using a biotin RNA Labeling Kit (R7061M, Beyotime, China). The transcribed RNA was incubated with total RNA extracts from HuCCT1 cells at 4°C overnight and then purified with streptavidin magnetic beads (HY‐K0208, MedChemExpress, USA). After extensive washing, the RNA complexes bound to the beads were eluted, and the level of MTX1 mRNA in the eluates was detected by PCR detection. The primers used are as follows:

Sense NKILA‐F:
TAATACGACTCACTATAGGGAGACCCGGCACCCGCGCAACGGAG
Sense NKILA‐R: TCCAGTTAAATTGAGATATACTTA
Antisense NKILA‐F:
TAATACGACTCACTATAGGGTCCAGTTAAATTGAGATATACTTA
Antisense NKILA‐R: AGACCCGGCACCCGCGCAACGGAG

### 2.14. RNA Stability Assay

HuCCT1 cells were transfected with OE‐negative control (NC) or OE‐NKILA. Then cells (2.5 × 10^5^ cells/well) were inoculated in 6‐well plates and cultured overnight, followed by incubation with actinomycin D (SBR00013, Sigma‐Aldrich, USA) for 0, 2, 4, 6, 8, 10, and 12 h to measure RNA stability. Total RNA was extracted at each time point after treatment and qRT‐PCR was performed to quantify MTX1 mRNA.

### 2.15. Co‐IP

Pierce Classic Magnetic Bead Assay IP/Co‐IP Kit (88804, Thermo Scientific, USA) was used to detect the binding of MTX1 and TOMM40. HuCCT1 cells transfected with knockdown‐NC (KD‐NC) or KD‐NKILA were incubated for 5 min with precooled IP buffer, and centrifuged to obtain supernatants. Cell lysates were conjugated with 2–10 μg immunoprecipitating antibodies (NC IgG) (A7016, Beyotime, China), MTX1 (#29890, 1/200, CST, USA), and TOMM40 (ab185543, 1/50, Abcam, UK) overnight at 4°C, and cultivated with magnetic beads for 1 h at room temperature. The antigen/antibody complexes were eluted, followed by detection of Western blot on the amount of MTX1 and TOMM40 proteins in each group.

### 2.16. Immunofluorescence

Transfected cells were fixed for 30 min, incubated for 30 min using MTX1 (PA5‐119279, 5 μg/mL, Thermo Fisher, USA), and TOMM40 (66658‐1‐Ig, 1/500, Proteintech, China) antibodies, and cultured with fluorescent secondary antibodies coupled to Alexa Fluor 647 (A0468, Beyotime, China) or Alexa Fluor 488 (A0428, Beyotime, China). The nuclei were stained using a 4′, 6‐diamidino‐2‐phenylindole (DAPI) (C1005, Beyotime, China) working solution. Afterwards, the location of MTX1 and TOMM40 expressions was visualized under a fluorescence microscope (200×).

### 2.17. Cell Counting Kit‐8 (CCK‐8) Assay

After HuCCT1 cells were transfected with OE‐MTX1 and/or KD‐NKILA/KD‐TOMM40, cell viability was determined using the CCK‐8 kit (C0037, Beyotime, China). Briefly, each group of cells (2 × 10^3^) was cultured for 24 h and additionally cultured with 10 μL CCK‐8 solution for 2 h. The absorbance at 450 nm was detected using a microplate reader.

### 2.18. Transwell Assay

Cells in each group were treated as before, and Transwell chambers (CLS3384, Merck, Germany) as well as matrix gel (HY‐K6002, MCE, USA) was used for invasion assay determination. Cells cultured in serum‐free medium were added to the upper chamber of the Transwell (lined with matrix gel), while medium with 10%–20% FBS was added to the lower layer to form a nutrient gradient. About 24 h later, excess cells were removed, and invading cells were fixed and stained with crystal violet (HY‐B0324A, MCE, USA) and observed under a microscope (200×).

### 2.19. Flow Cytometry

Single‐cell suspensions were prepared by digesting tumor tissues using proteinase K (ST532, Beyotime, China), and then washed with staining buffer. Cells were stained with FITC‐labeled CD45 (0.5 µg, 11‐0451‐82, Invitrogen, USA) or PE‐labeled CD8 (0.1 µg, MA5‐17849, Invitrogen, USA) for 30 min at 4°C. Afterwards, CD8^+^ T cell percentage was analyzed using a FACSCalibur flow cytometer (BD Biosciences, USA) and FlowJo v.10 software (FlowJo, USA).

### 2.20. Mouse Tumor Model

C57BL/6 mice (*n* = 50, 4 weeks old, male) were purchased from Hangzhou Medical College and reared under SPF conditions according to local institution guidelines. After subcostal dissection, 5 × 10^6^/mL (0.2 mL) stably transfected HuCCT1 or RBE cells mixed with PBS and matrix gel solution were injected subcutaneously into the mice [22]. Tumor size was measured with vernier calipers every 3 days, and continued to be observed until day 24. At the end of the last observation, the mice were euthanized by cervical dislocation after anesthesia (sodium pentobarbital, STY667, Zzsiji, China) and the tissues were collected.

### 2.21. Statistical Analysis

Statistical analysis was performed by Graphpad 8.0 software (Graphpad Prism, USA). Two‐group comparison was performed by independent sample *t*‐test. For comparisons among mutiple groups, one‐way or two way analysis of variancewas used; for multiple contrasts, Dunnett’s test or Tukey’s test was conducted. A *p*  < 0.05 was defined as statistically significant.

## 3. Results

### 3.1. Differential Expression of NKILA in ICC and Association Analysis With Warburg Effect

Based on the UALCAN analysis, we found that NKILA, MTX1, and TOMM40 were highly expressed in cholangiocarcinoma (Figure 1A). MTX1, a mitochondrial protein, may be implicated in the Warburg process, a metabolic characteristic of cancers [11]. We discovered that tumor tissues expressed considerably more Glut1, LDH, and PKM2 than adjacent tissues, while LC3B/LC3A level was decreased. (Figure 1B–D, *p*  < 0.05). Meanwhile, high NKILA levels were detected in tumor tissues (Figure 1E, *p*  < 0.01). ICC cells RBE, HCCC‐9810, and HuCCT1 showed significantly higher NKILA expression compared to HIBEpic cells (Figure 1F, *p*  < 0.01). Accordingly, the next studies focused on HuCCT1, which expressed the most NKILA.

Figure 1Expression profiles of NKILA, MTX1, TOMM40, and key proteins related to Warburg effect and autophagy in ICC tissues and cell lines. (A) UALCAN analyzed the expressions of NKILA, MTX1, and TOMM40. (B–D) Immunohistochemistry (IHC) was performed to detect glucose transporter type 1 (Glut1), lactate dehydrogenase (LDH), pyruvate kinase isozyme typeM2 (PKM2), and light chain 3 (LC3) expressions in tumor tissues (*N* = 10) (scale: 50 μm). (E) The expression of NKILA in tumor tissues was detected by qRT‐PCR. (F) The expression level of NKILA in HIBEpic, REB, HCCC‐9810, and HuCCT1 cells were detected by qRT‐PCR. The internal parameter is β‐actin. Each experiment was repeated three times.  ^∗^ *p* < 0.05,  ^∗∗^ *p* < 0.01,  ^∗∗∗^ *p* < 0.001.

**Figure:**
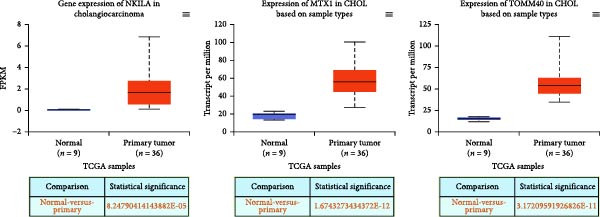
(A)

**Figure:**
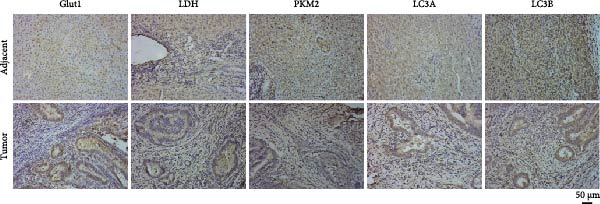
(B)

**Figure:**
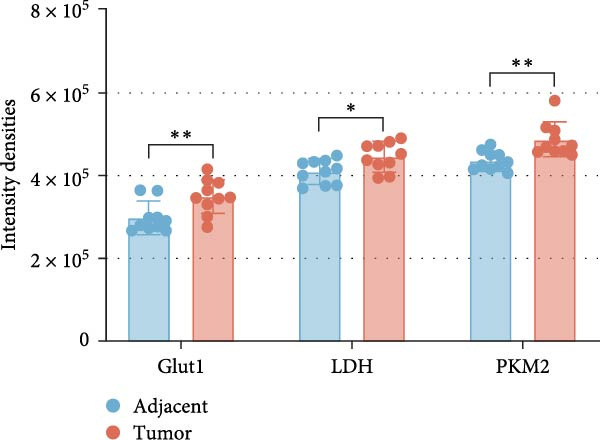
(C)

**Figure:**
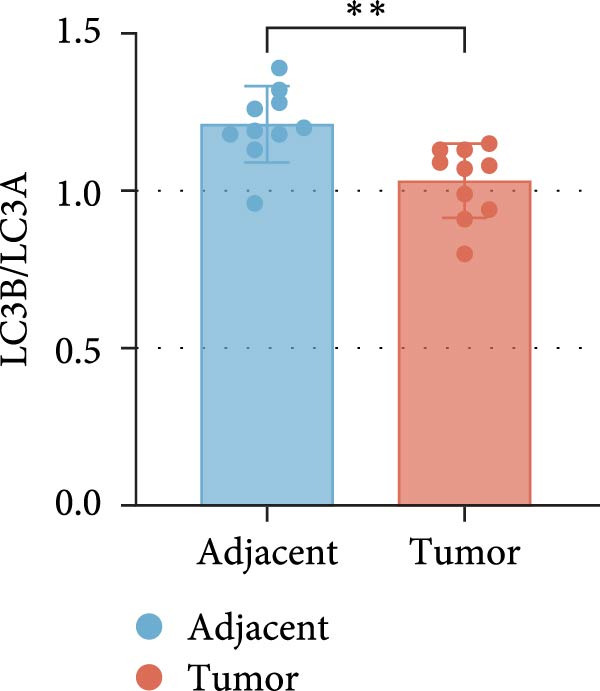
(D)

**Figure:**
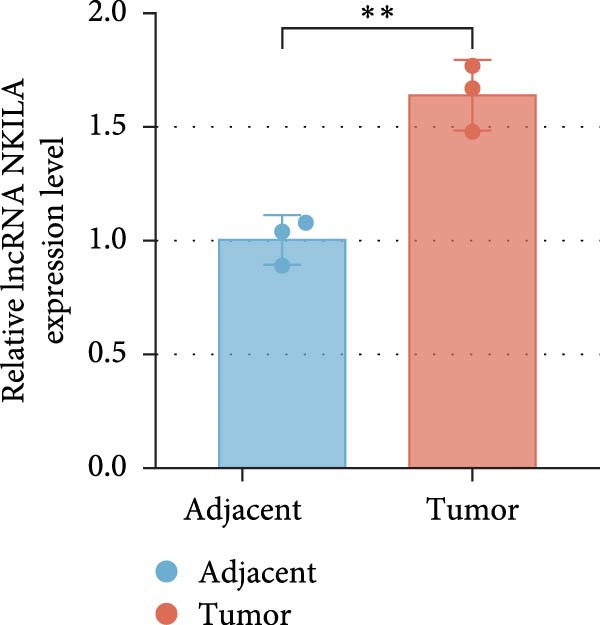
(E)

**Figure:**
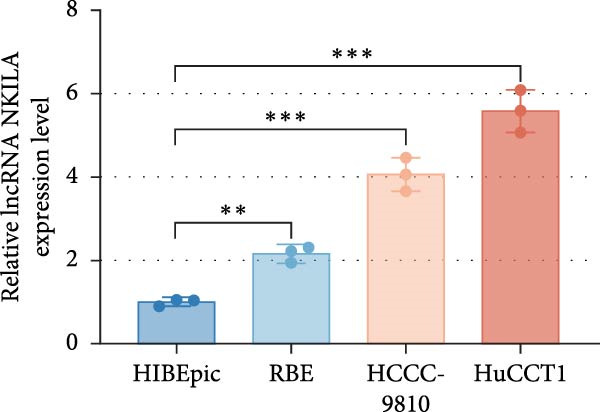
(F)

### 3.2. NKILA Affected ICC Cells Through the Warburg Effect and Autophagy In Vitro

Warburg effect inhibitor DCA was employed in rescue tests. It was shown that NKILA expression was suppressed by NKILA knockdown and enhanced by NKILA overexpression (Figure 2A, *p*  < 0.001). NKILA silencing decreased EdU‐positive cells and triggered apoptosis, while NKILA overexpression exerted the opposite effects; also, DCA offset the regulation of NKILA overexpression (Figure 2B,C, *p*  < 0.05). NKILA silencing attenuated epithelial–mesenchymal transition (EMT), decreased N‐cadherin and vimentin levels (Figure 2D, Supporting Information 1: Figure S1A,C, *p*  < 0.05), and increased E‐cadherin level (Figure 2D, Supporting Information 1: Figure S1B, *p*  < 0.001), but NKILA overexpression played an opposite role. DCA could reverse the effect of NKILA overexpression (Figure 2D, Supporting Information 1: Figure S1A,C, *p*  < 0.05), but had no significant effect on E‐cadherin (Figure 2D, Supporting Information 1: Figure S1B).

Figure 2NKILA silencing inhibited cell growth, epithelial–mesenchymal transition (EMT), Warburg effect, autophagy, and enhanced CD8^+^ T cell toxicity of HuCCT1 cells. (A) The transfection efficiency of NKILA was detected by qRT‐PCR. The internal parameter is β‐actin. (B) 5‐ethynyl‐2′‐deoxyuridine (EdU) assay was used to detect cell proliferation in each group (scale: 40 μm). (C) Terminal dexynucleotidyl transferase (TdT)‐mediated dUTP nick end labeling (TUNEL) was used to detect apoptosis in each group (scale: 40 μm). (D) Western blot was used to detect the expressions of EMT‐related proteins N‐cadherin, E‐cadherin, and vimentin. (E) Western blot was conducted to measure the expressions of Warburg‐related proteins Glut1, hexokinase2 (HK2), PKM2, LDH, and monocarboxylate transporter 1 (MCT1). (F) The effects of NKILA silencing on glucose uptake (scale: 40 μm), pyruvate levels, lactate levels, adenosine triphosphate levels (ATP), pyruvate kinase (PK), and LDH were determined using kits. (G) Western blot was used to examine the expressions of autophagy‐related proteins sequestosome 1 (P62), Beclin1, and LC3. (H) Western blot was conducted to examine the expressions of mammalian target of rapamycin (mTOR) pathway and programed cell death 1 ligand 1 (PD‐L1)‐related proteins. (I) LDH cytotoxicity assay was performed to measure the toxic effects of CD8^+^ T cells. Each experiment was repeated three times.  ^∗^ *p* < 0.05,  ^∗∗^ *p* < 0.01,  ^∗∗∗^ *p* < 0.001.

**Figure:**
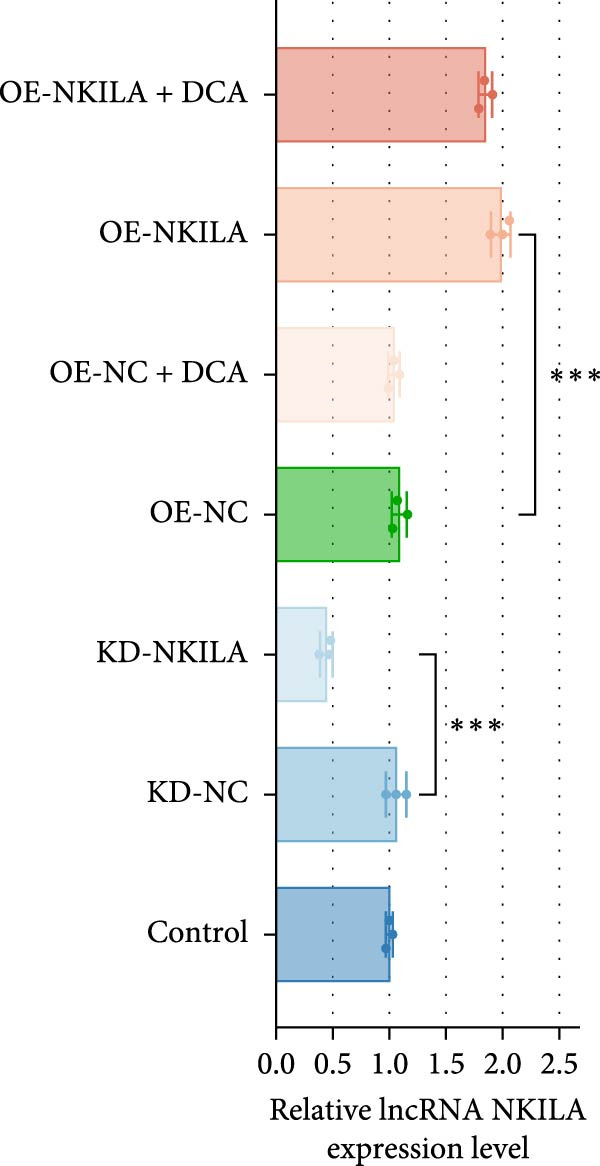
(A)

**Figure:**
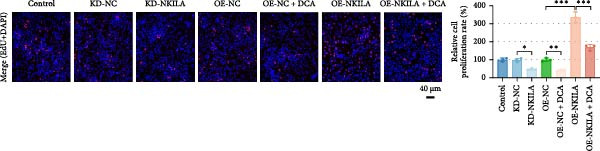
(B)

**Figure:**
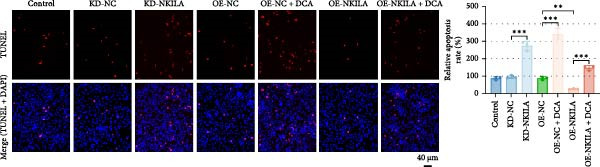
(C)

**Figure:**
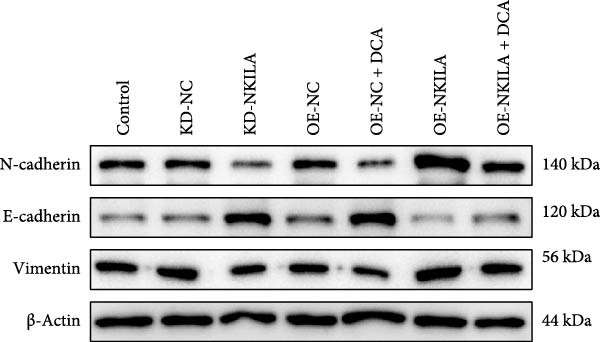
(D)

**Figure:**
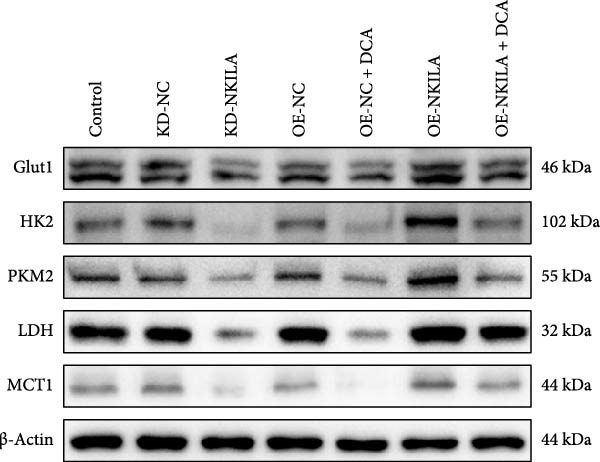
(E)

**Figure:**
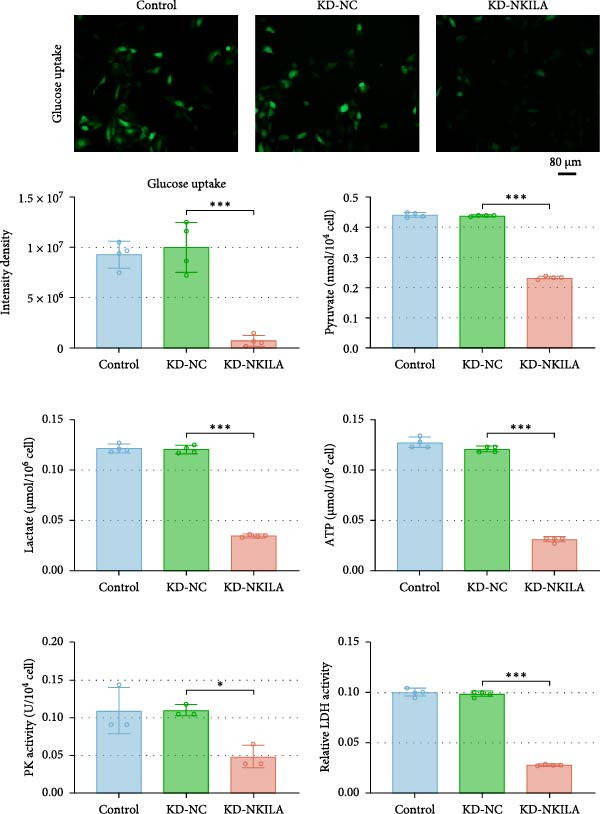
(F)

**Figure:**
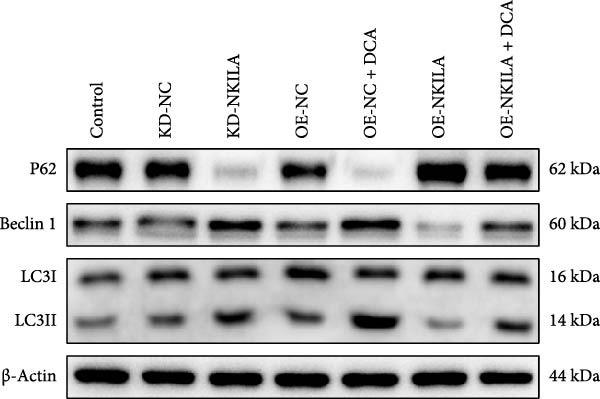
(G)

**Figure:**
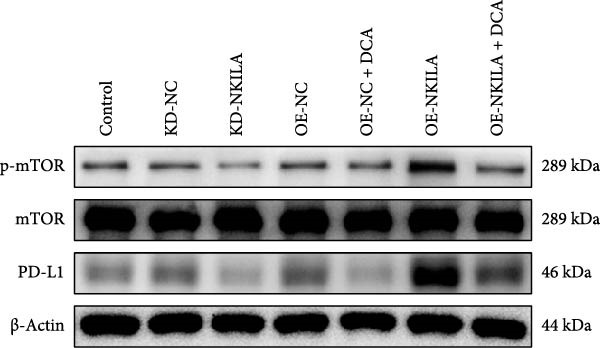
(H)

**Figure:**
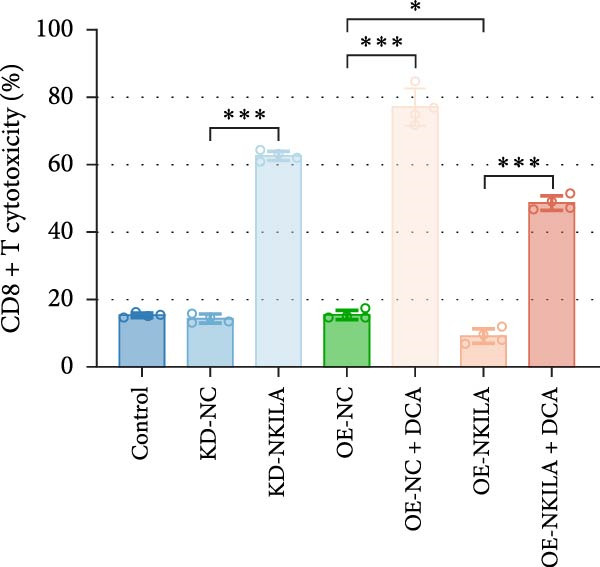
(I)

Next, we examined Warburg effect‐related proteins and markers, including Glut1, HK2, PKM2, LDH, and MCT1. NKILA knockdown or DCA treatment downregulated Glut1, HK2, PKM2, LDH, and MCT1 protein levels, while NKILA overexpression promoted the expression of these proteins (Figure 2E, Supporting Information 1: Figure S1D–H, *p*  < 0.001). DCA weakened the promoting role of NKILA overexpression (Figure 2E, Supporting Information 1: Figure S1D–H, *p*  < 0.001). Additionally, NKILA silencing decreased glucose uptake, pyruvate levels, lactate production, ATP levels, and PK and LDH activities (Figure 2F, *p*  < 0.05). As indicated, Warburg‐related protein PKM2 activated the mTOR pathway, which can suppress autophagy. When autophagy occurs, the level of PD‐L1 in cells also decreases. Figure 2G,H showed that NKILA silencing (compared to KD‐NC) or DCA (compared to OE‐NC) decreased P62, inhibited p‐mTOR/mTOR and PD‐L1, and raised Beclin 1 and LC3II/I (Supporting Information 1: Figure S1I–M, *p*  < 0.05). DCA reversed the impacts of NKILA overexpression on the above proteins (Figure 2G,H, Supporting Information 1: Figure S1I–M, *p*  < 0.001). NKILA knockdown increased, while NKILA overexpression diminished CD8^+^ T‐cell toxicity, and DCA reversed the effect of NKILA overexpression (Figure 2I, *p*  < 0.05).

### 3.3. NKILA Modulated MTX1 mRNA Stability and MTX1‐TOMM40 Combination

ENCORI was used to examine NKILA and MTX1 binding sites (Figure 3A), which is located within the 162–199 bp region of the NKILA sequence. Clinical sample validation showed that MTX1 and TOMM40 expressions were higher in tumor tissues than adjacent tissues (Figure 3B,C, *p*  < 0.01). NKILA silencing decreased MTX1 expression, while NKILA overexpression had the opposite impact (Figure 3D,E, Supporting Information 1: Figure S1N, *p*  < 0.01). Additionally, we confirmed RNA‐RNA duplex formation between NKILA and MTX1 (Figure 3F–I, *p*  < 0.001). NKILA and MTX1 binding was verified by RIP and PCR (Figure 3J,K, *p*  < 0.01). Then the combination of NKILA and MTX1 was further validated by RNA–RNA pull‐down assay (Figure 3L,M, *p*  < 0.001). To validate whether NKILA interacts with MTX1 at the predicted binding site illustrated in Figure 3A, we constructed NKILA mutants: truncated‐NKILA‐1 (which contains the predicted binding sequence), truncated‐NKILA‐2 (which lacks the predicted binding sequence), and Mut‐NKILA (which carries mutations in the predicted sequence). RNA–RNA pull‐down assays demonstrated that the NKILA site illustrated in Figure 3A is essential for its interaction with MTX1 (Figure 3N,O, *p*  < 0.01). In addition, actinomycin D treatment showed that NKILA stabilized MTX1 mRNA (Figure 3P).

Figure 3NKILA, MTX1, and TOMM40 interact with each other. (A) ENCORI analyzed the binding sites of NKILA and MTX1. (B, C) qRT‐PCR detected the expressions of MTX1 and TOMM40 in ICC tissues. The internal parameter is β‐actin. (D, E) The effect of NKILA silencing or overexpression on MTX1 expression was detected by qRT‐PCR and Western blot. The internal parameter is β‐actin. (F–I) Ribonuclease protection assay (RPA) detected RNA‐RNA double‐stranded body formation. (J, K) RNA immunoprecipitation (RIP) and PCR detected the binding of NKILA to MTX1. (L, M) RNA‐RNA pull down detected the binding of NKILA to MTX1. (N, O) RNA‐RNA pull down detected the bind sites of NKILA to MTX1. NKILA sense: full‐length sense strand of NKILA; truncated‐NKILA‐1: contains the binding sequence predicted in Figure 3A; truncated‐NKILA‐2: lacks the binding sequence predicted in Figure 3A; Mut‐NKILA: contains site‐specific mutations within the binding sequence predicted in Figure 3A. (P) The effect of NKILA on the stability of MTX1 was detected by qRT‐PCR. The internal parameter is β‐actin. (Q) Western blot was used to detect the expression of MTX1 in ICC cell lines. (R) Search tool for recurring instances of neighboring genes (STRING) was conducted to analyze the interaction proteins of MTX1. (S) The effect of NKILA silencing on the binding of MTX1 and TOMM40 was detected using coimmunoprecipitation (Co‐IP). Each experiment was repeated three times.  ^∗∗^ *p* < 0.01,  ^∗∗∗^ *p* < 0.001.

**Figure:**
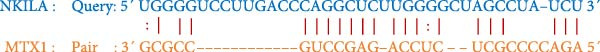
(A)

**Figure:**
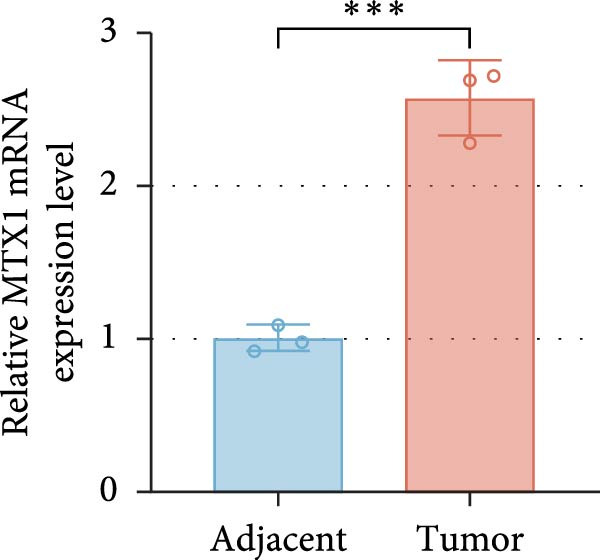
(B)

**Figure:**
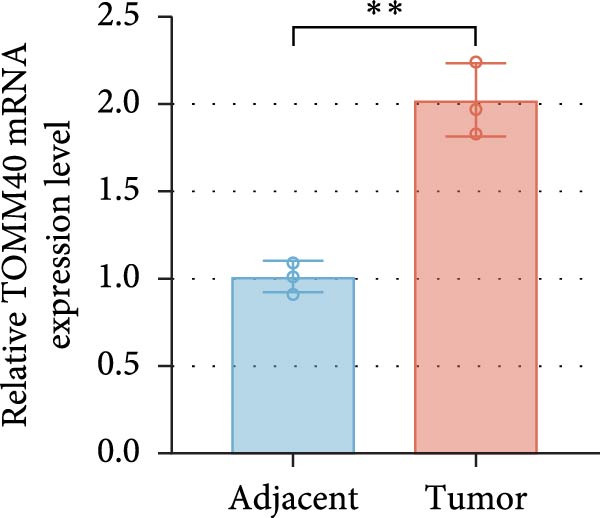
(C)

**Figure:**
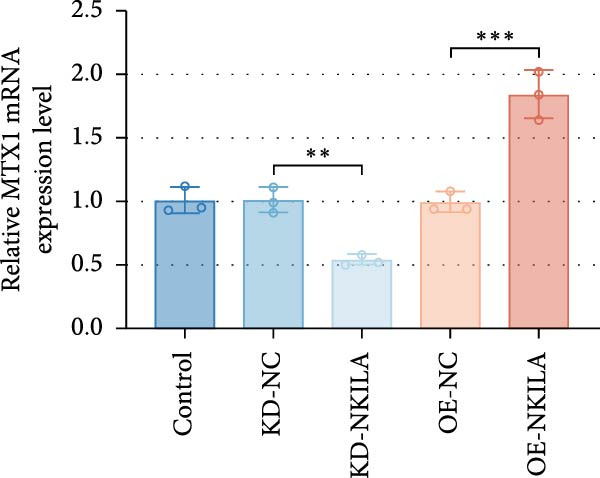
(D)

**Figure:**
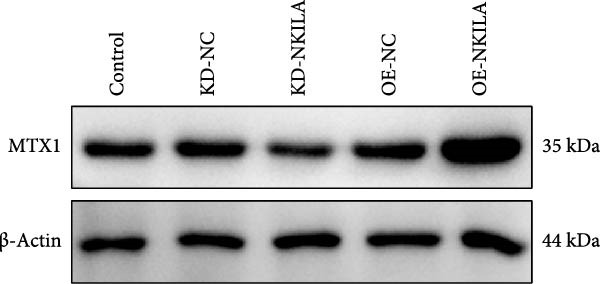
(E)

**Figure:**
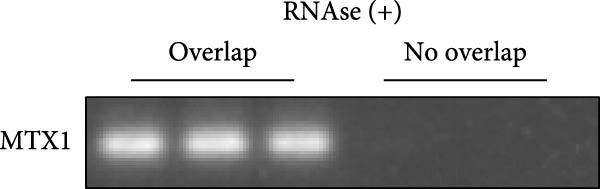
(F)

**Figure:**
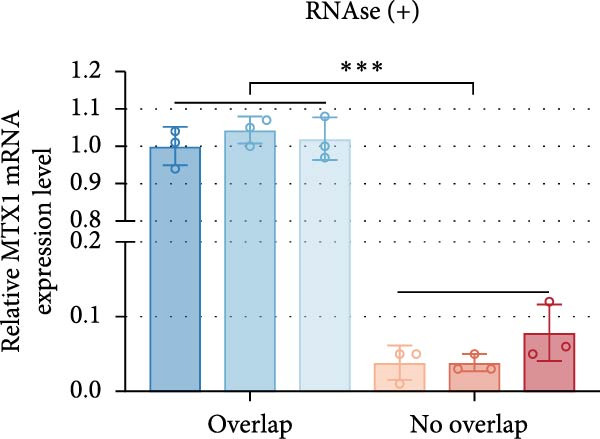
(G)

**Figure:**
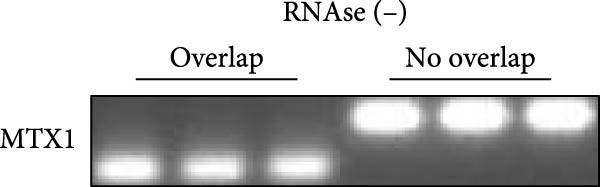
(H)

**Figure:**
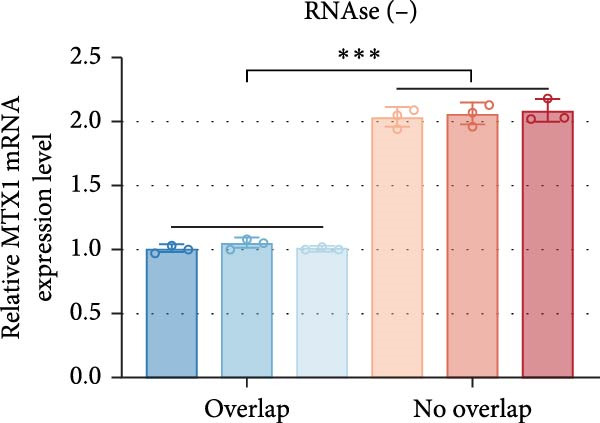
(I)

**Figure:**
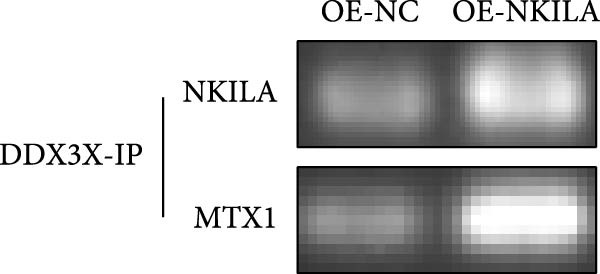
(J)

**Figure:**
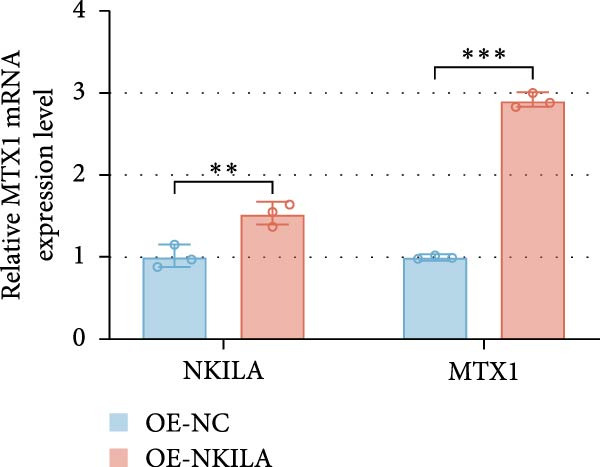
(K)

**Figure:**
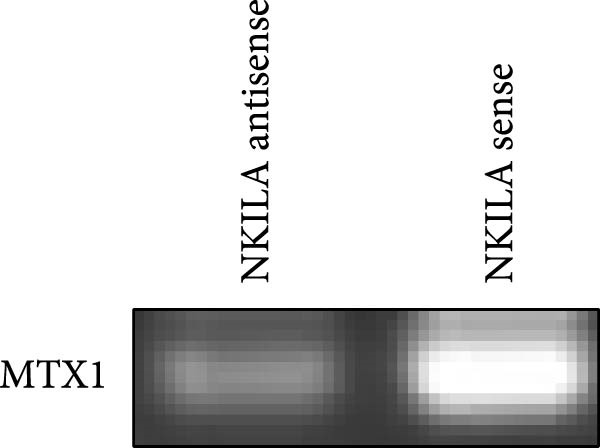
(L)

**Figure:**
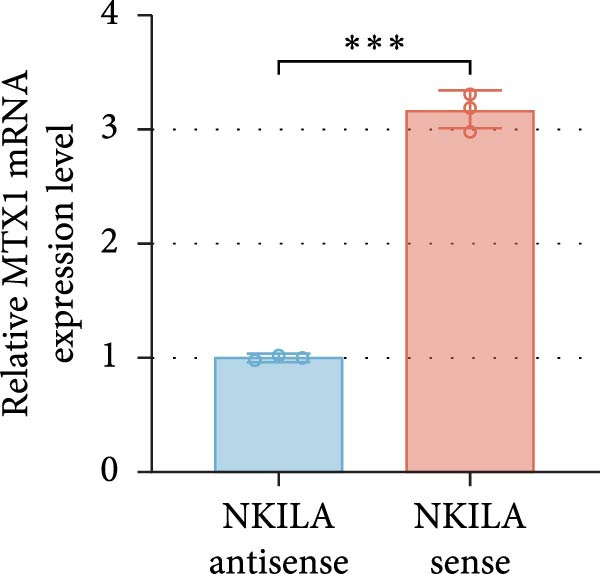
(M)

**Figure:**
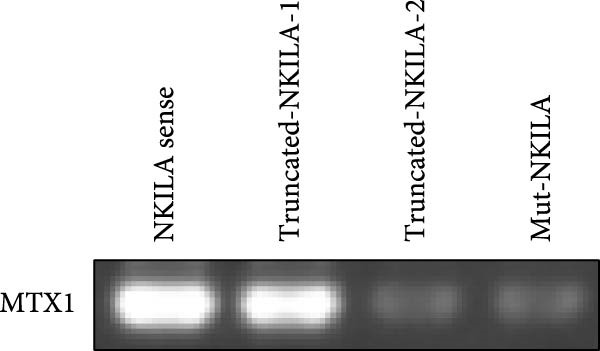
(N)

**Figure:**
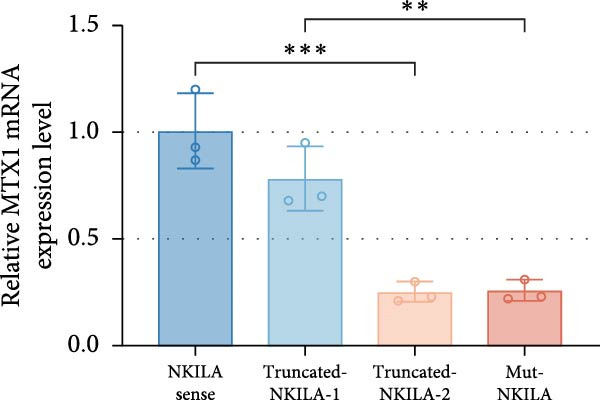
(O)

**Figure:**
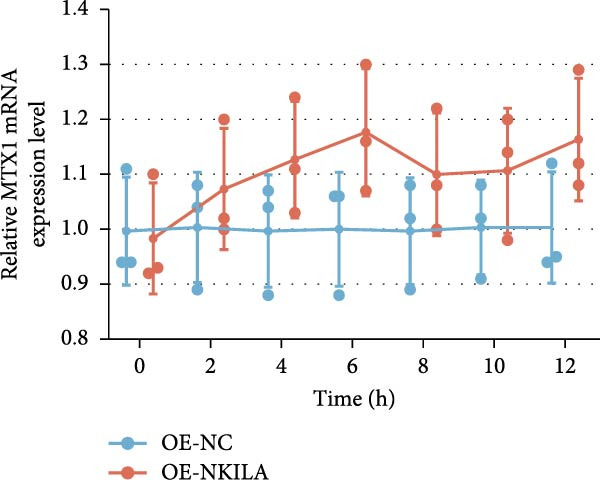
(P)

**Figure:**
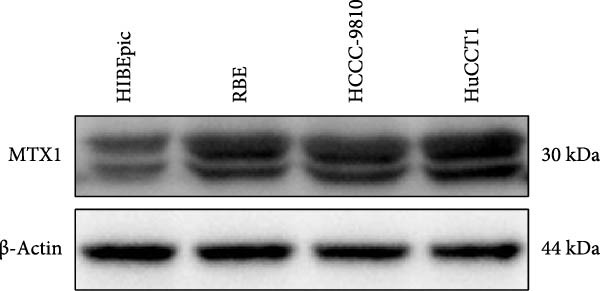
(Q)

**Figure:**
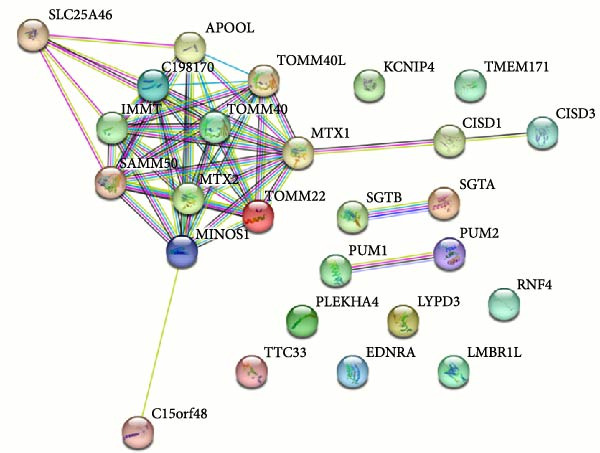
(R)

**Figure:**
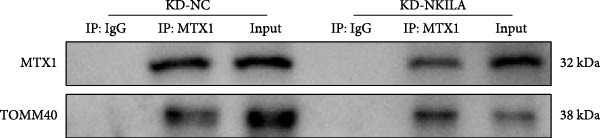
(S)

Cell test results revealed MTX1 expression was considerably greater in cholangiocarcinoma cells than HIBEpic cells (Figure 3Q, Supporting Information 1: Figure S1O, *p*  < 0.001). The interacting proteins of MTX1 were examined using STRING (Figure 3R). MTX1 bound to TOMM40 as confirmed in Co‐IP studies, while NKILA silencing reduced MTX1 binding to TOMM40 (Figure 3S). MTX1 and TOMM40 did not affect NKILA expression according to qRT‐PCR assays results (Figure 4A). NKILA knockdown reduced yet MTX1 overexpression promoted MTX1 and TOMM40 expressions, and KD‐TOMM40 exerted suppressing effects (Figure 4B, Supporting Information 1: Figure S1P,Q, *p*  < 0.001). Immunofluorescence analysis outcomes revealed colocalization of TOMM40 and MTX1, and NKILA/TOMM40 knockdown reversed the increasing fluorescence intensity of MTX1 and TOMM40 by MTX1 overexpression (Figure 4C, *p*  < 0.05).

Figure 4NKILA knockdown inhibited the expression and colocalization of MTX1 and TOMM40, and MTX1 overexpression reversed the regulatory effects of NKILA silencing on cell growth. (A) The effects of MTX1 and TOMM40 on the expression of NKILA was detected by qRT‐PCR. The internal parameter is β‐actin. (B) The expressions of MTX1 and TOMM40 proteins in each group was detected by Western blot. (C) Immunofluorescence was performed to detect the location of MTX1 and TOMM40 in each group (scale: 40 μm). (D) Cell Counting Kit‐8 (CCK‐8) assay was carried out to detect the effects of NKILA/TOMM40 silencing or MTX1 overexpression on cell viability. (E) TUNEL was used to test apoptosis (scale: 40 μm). Each experiment was repeated three times.  ^∗^ *p* < 0.05,  ^∗∗^ *p* < 0.01,  ^∗∗∗^ *p* < 0.001.

**Figure:**
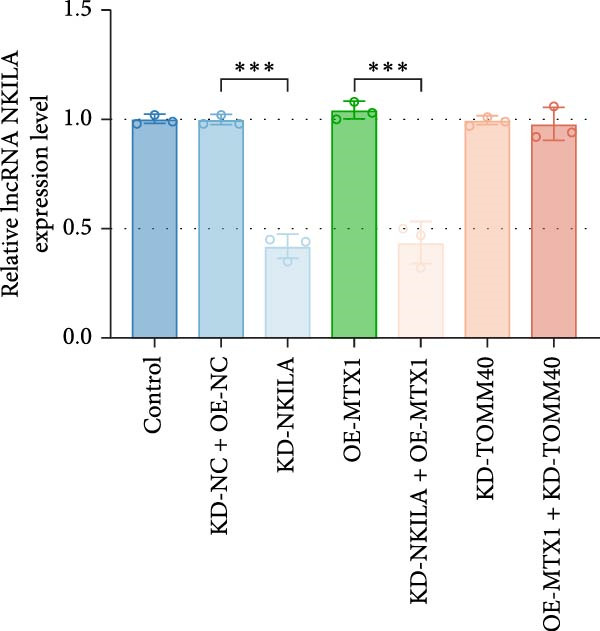
(A)

**Figure:**
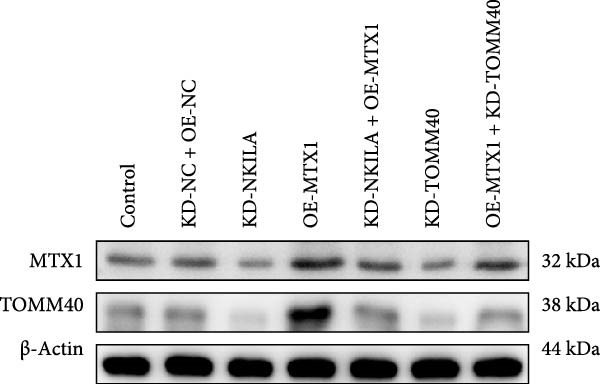
(B)

**Figure:**
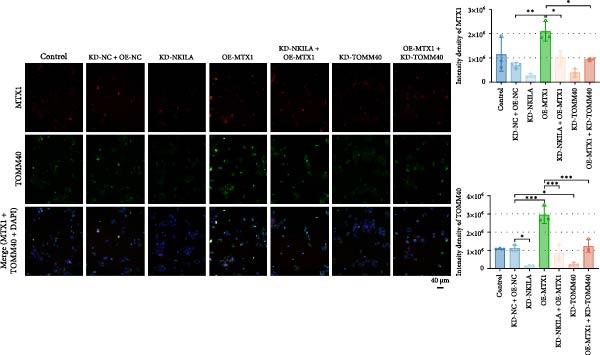
(C)

**Figure:**
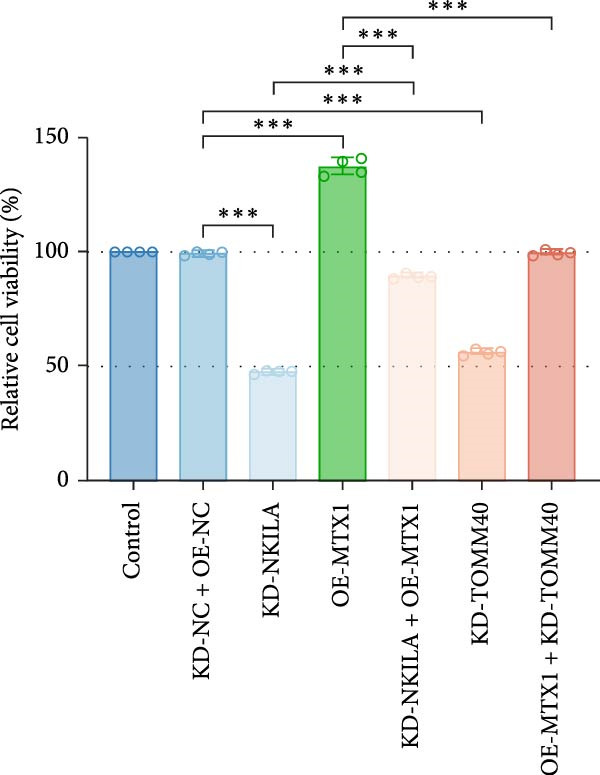
(D)

**Figure:**
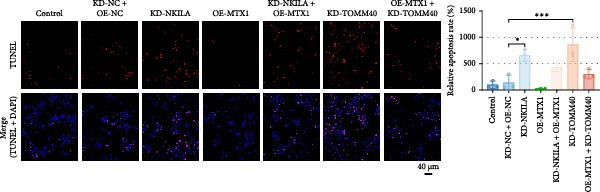
(E)

### 3.4. NKILA Mediated Warburg Effect and Autophagy Through MTX1‐TOMM40 to Modulate ICC Progression

Functional experiment data showed that NKILA/TOMM40 silencing reversed the promoting effect of MTX1 overexpression on cell viability (Figure 4D, *p*  < 0.001). Both NKILA and TOMM40 silencing induced cell apoptosis (Figure 4E, *p*  < 0.05), overexpression of MTX1 inhibited apoptosis but the effect was not significant. Functional investigations demonstrated that NKILA/TOMM40 silencing abrogated MTX1 overexpression‐induced EMT protein regulation, inhibited N‐cadherin and vimentin, and increased E‐cadherin expression (Figure 5A, Supporting Information 1: Figure S1R–T, *p*  < 0.05). Similar results were also detected in Transwell experiments, which unveiled that MTX1 overexpression caused cell invasion, whereas NKILA/TOMM40 knockdown reversed its role (Figure 5B, *p*  < 0.05). NKILA silencing‐inhibited Glut1, HK2, PKM2, LDH, and MCT1 protein expressions were upregulated by MTX1 overexpression, which was further reversed by TOMM40 silencing (Figure 5C, Supporting Information 2: Figure S2A–E, *p*  < 0.001). NKILA/TOMM40 silencing offset the promoting role of MTX overexpression in glucose uptake, pyruvate, lactate production, ATP levels, and PK and LDH activities and its suppressing role in the Warburg effect (Figure 5D, *p*  < 0.01).

Figure 5MTX1 overexpression reversed the regulatory effects of NKILA silencing on EMT proteins, cell invasion, Warburg effect, autophagy, and CD8^+^ T cell toxicity. (A) Western blot was performed to examine the expressions of EMT‐related proteins. (B) The effects of NKILA/TOMM40 silencing and MTX1 overexpression on cell invasion were analyzed by Transwell assay (scale: 100 μm). (C) Western blot was conducted to quantify the expressions of Warburg‐associated proteins. (D) Warburg effect‐related indicators (glucose uptake [scale: 50 μm], pyruvate, lactate, ATP, PK, and LDH) were examined. (E) Western blot was applied to measure the expressions of autophagy‐related proteins. (F) Western blot was employed to detect the expressions of mTOR pathway‐ and PD‐L1‐related proteins. (G) LDH cytotoxicity assay was performed to determine the toxic effects of CD8^+^ T cells. Each experiment was repeated three times.  ^∗∗^ *p* < 0.01,  ^∗∗∗^ *p* < 0.001.

**Figure:**
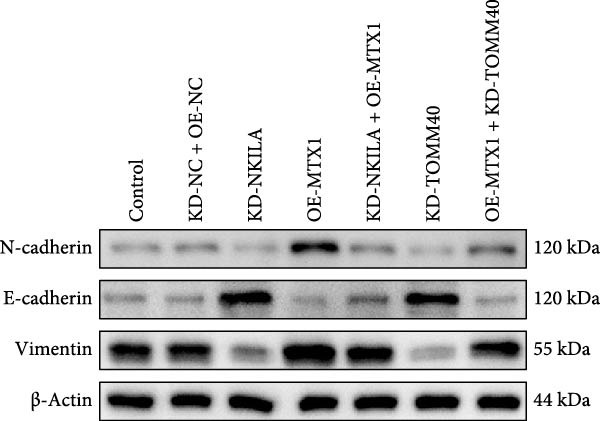
(A)

**Figure:**
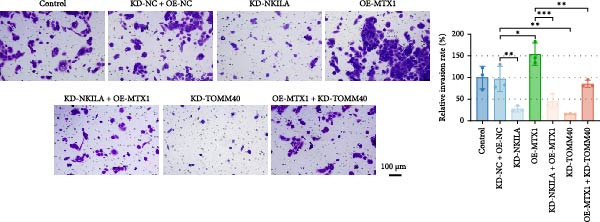
(B)

**Figure:**
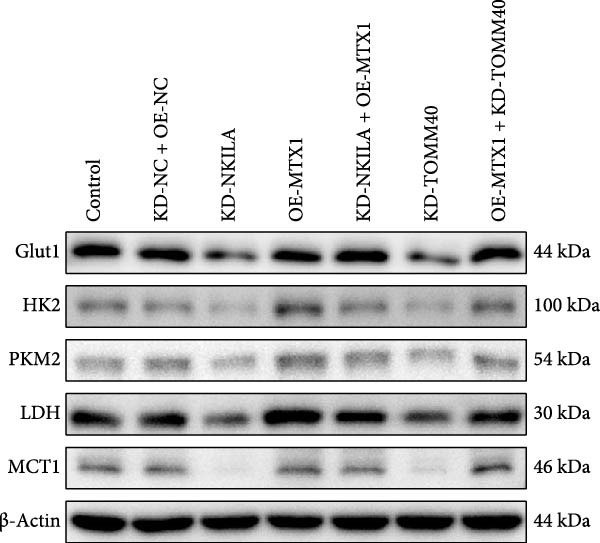
(C)

**Figure:**
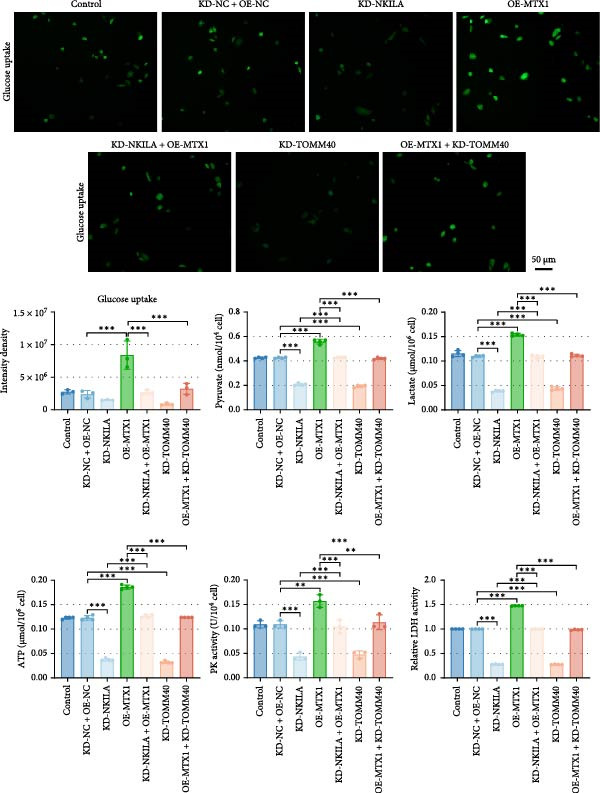
(D)

**Figure:**
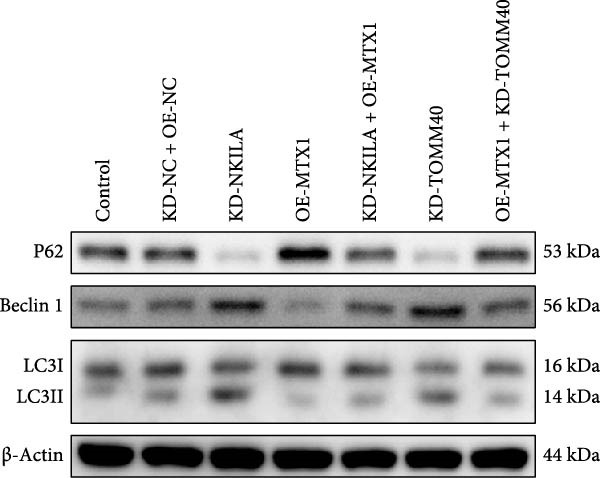
(E)

**Figure:**
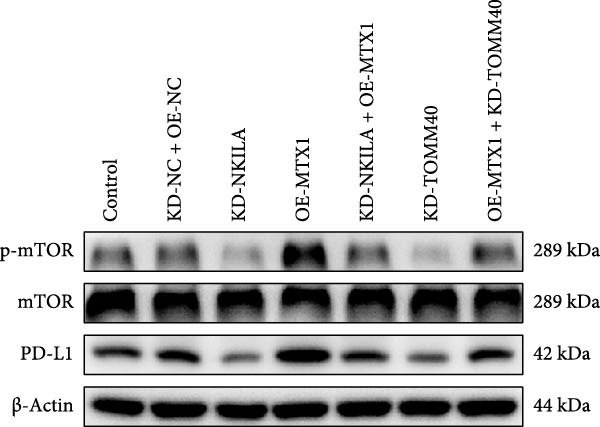
(F)

**Figure:**
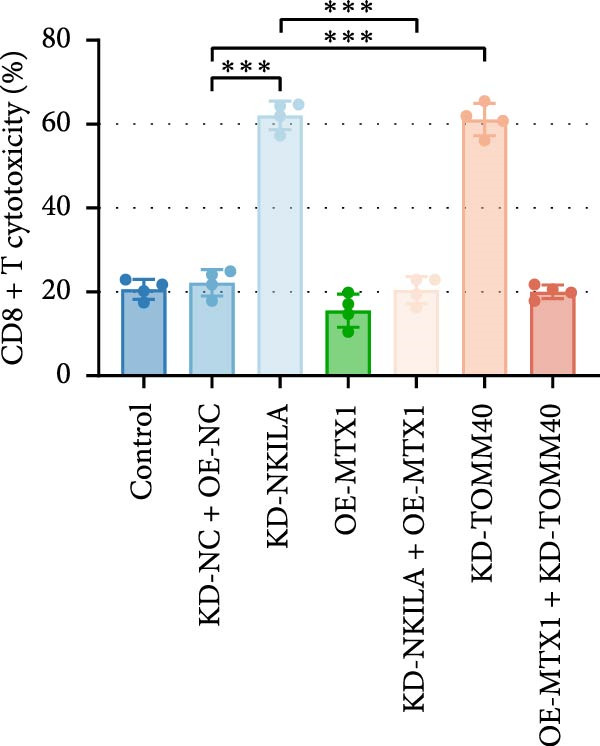
(G)

Autophagy was enhanced by NKILA silencing (as evidenced by upregulation of Beclin 1 and LC3II/I and downregulation of P62), yet hindered by MTX1 overexpression, and TOMM40 silencing reversed the impact of MTX1 overexpression (Figure 5E, Supporting Information 2: Figure S2F–H, *p*  < 0.001). NKILA/TOMM40 silencing can block mTOR signaling pathway activated by MTX1 overexpression and downregulated PD‐L1 expression level (Figure 5F, Supporting Information 2: Figure S2I,J, *p*  < 0.001). NKILA/TOMM40 silencing increased CD8^+^ T cytotoxicity (Figure 5G, *p*  < 0.001), but MTX1 overexpression reversed the impact of NKILA silencing (Figure 5G, *p*  < 0.001).

### 3.5. In Vivo Experiments Detected the Effects of NKILA and MTX1 on Tumors

Next, we constructed a mouse ICC model using HuCCT1 (Figure 6) or RBE cells (Supporting Information 3: Figure S3) and found that NKILA silencing inhibited tumor growth compared to KD‐NC + OE‐NC, which was reversed by MTX1 overexpression (Figure 6A,B, Supporting Information 3: Figure S3A–C, *p*  < 0.05). Flow cytometry data suggested that MTX1 overexpression counteracted the promoting role of NKILA silencing in mouse CD8^+^ T cells in vivo (Figure 6C,D, Supporting Information 3: Figure S3D,E, *p*  < 0.001). NKILA silencing inhibited NKILA mRNA, and MTX1 and TOMM40 protein levels, while MTX1 overexpression reversed the inhibitory effect of NKILA silencing on MTX1 and TOMM40 (Figure 6E,F, Supporting Information 2: Figure S2K,L, Supporting Information 3: Figure S3F–I, *p*  < 0.001). Western blot and IHC experiment data exhibited that MTX1 overexpression abrogated NKILA silencing‐inhibited Warburg effect (Figure 6G–J, Supporting Information 2: Figure S2M–Q, Supporting Information 3: Figure S3J–L, *p*  < 0.05). Similarly, NKILA silencing induced autophagy, whilst MTX1 overexpression reversed the influence of NKILA silencing and inhibited mTOR signaling and PD‐L1 (Figure 6K,L, Supporting Information 2: Figure S2R–V, *p*  < 0.001).

Figure 6MTX1 overexpression reversed the improvement of NKILA silencing in mice with ICC, including Warburg effect and autophagy. (A, B) Mouse models of ICC were constructed and analyzed for tumor size. (C, D) Flow cytometry was performed to analyze the effects of MTX1 overexpression and NKILA silencing on the percentage of CD8^+^ T cells. (E) NKILA mRNA expression was detected by qRT‐PCR. The internal parameter is β‐actin. (F) MTX1 and TOMM40 protein levels were detected by Western blot. (G) Warburg‐related indices were determined by Western blot. (H–J) Warburg‐related indices were determined by IHC (scale: 100 μm). (K) Western blot detected autophagy‐related protein expression. (L) Western blot detected mTOR pathway and PD‐L1‐related protein expression. Each experiment was repeated three times.  ^∗∗^ *p* < 0.01,  ^∗∗∗^ *p* < 0.001.

**Figure:**
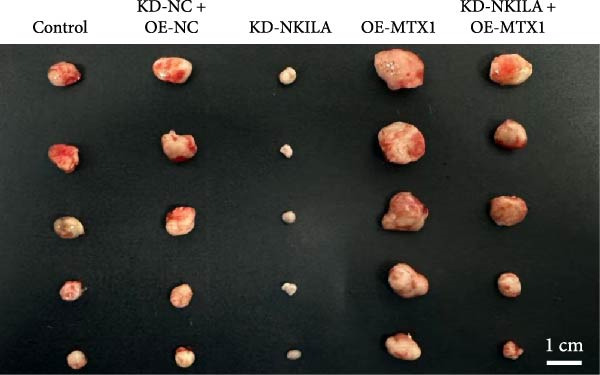
(A)

**Figure:**
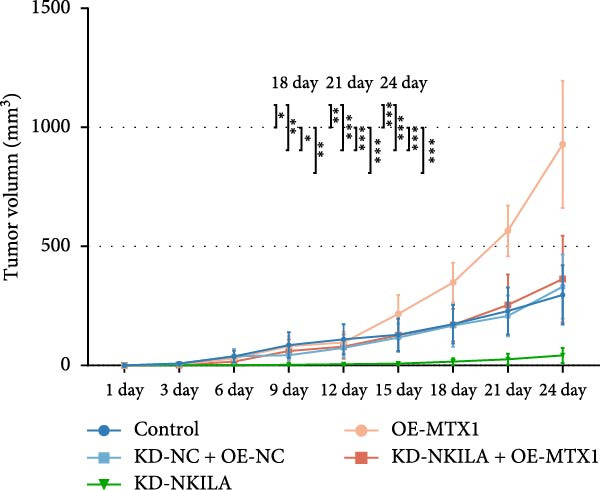
(B)

**Figure:**
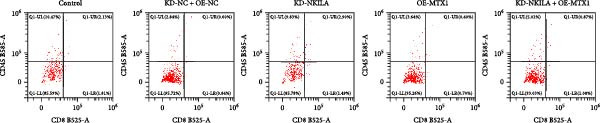
(C)

**Figure:**
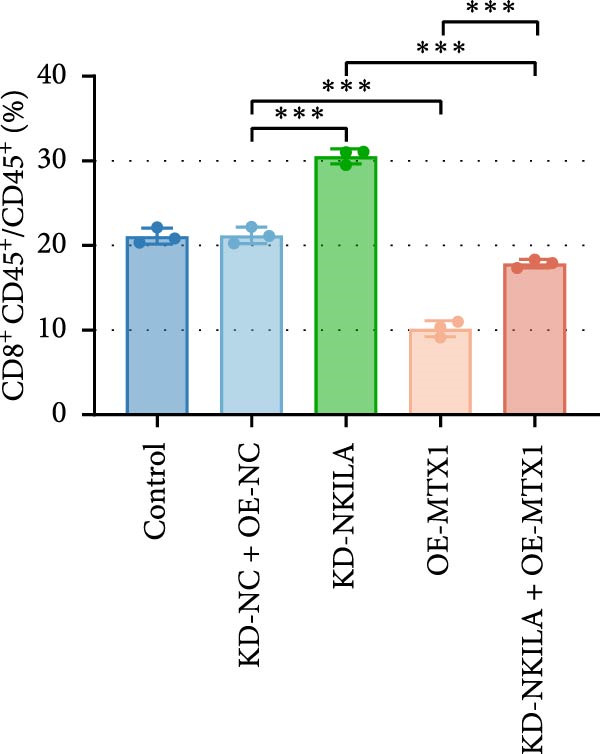
(D)

**Figure:**
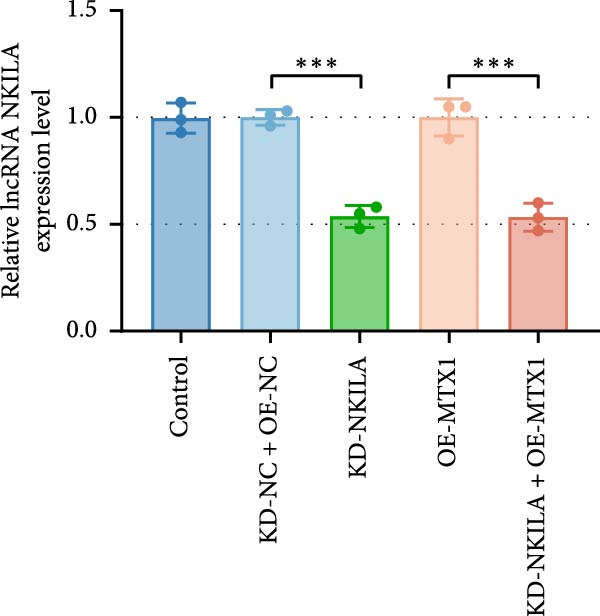
(E)

**Figure:**
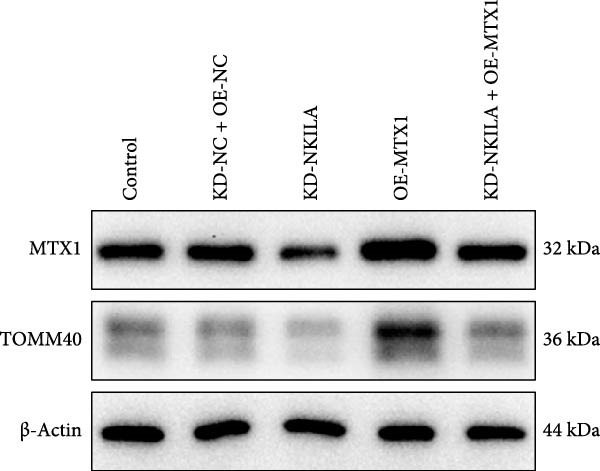
(F)

**Figure:**
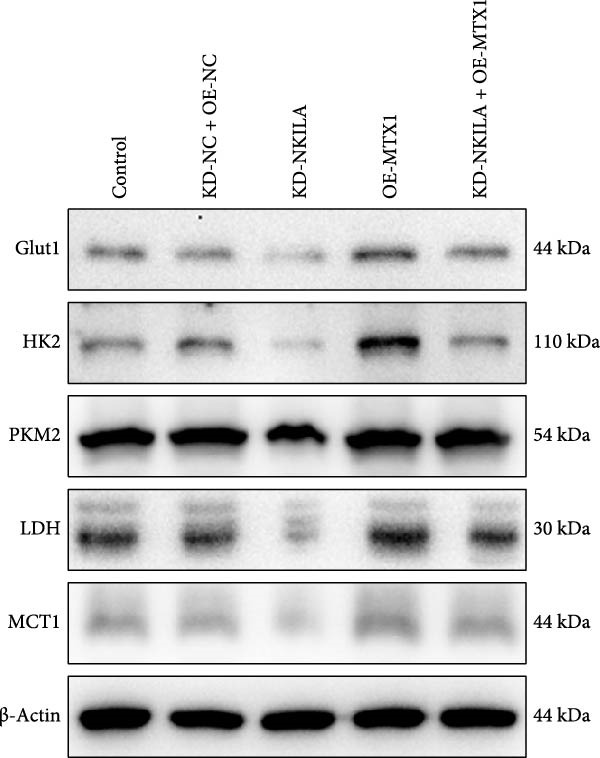
(G)

**Figure:**
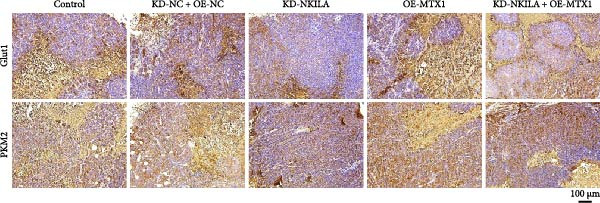
(H)

**Figure:**
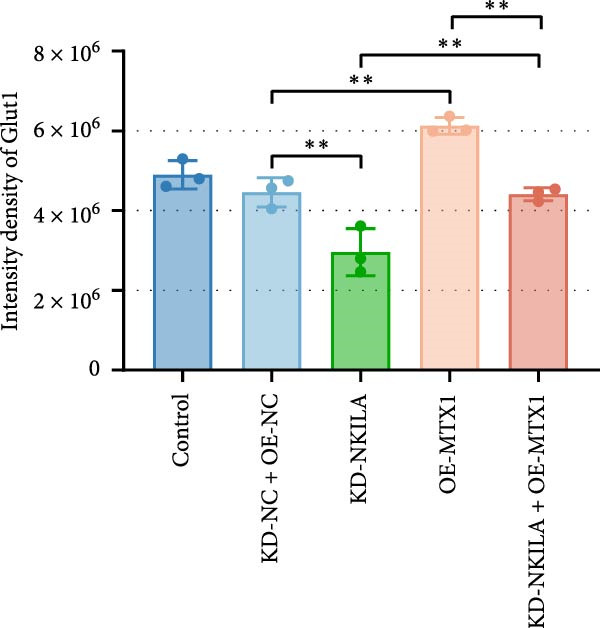
(I)

**Figure:**
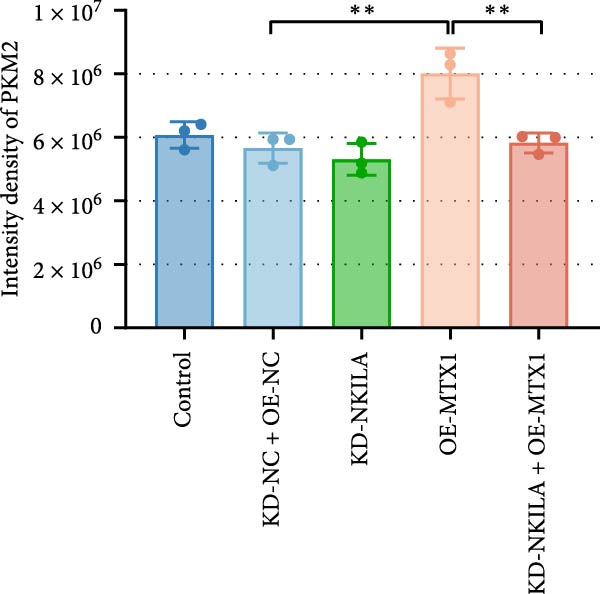
(J)

**Figure:**
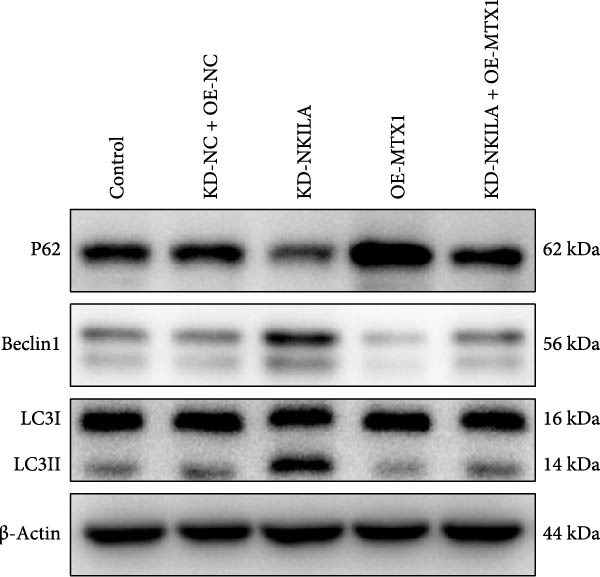
(K)

**Figure:**
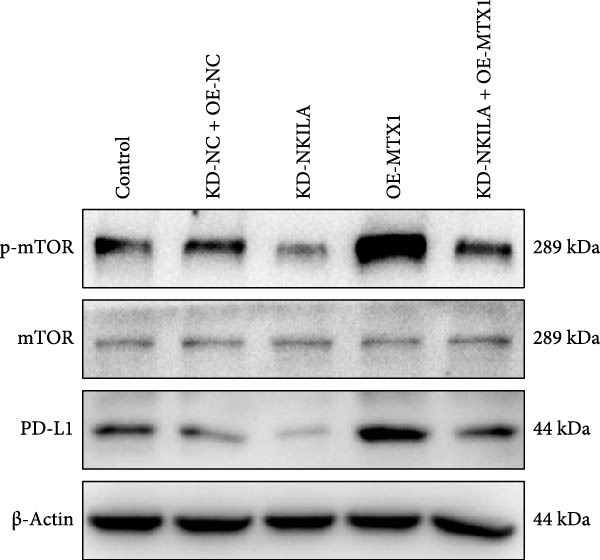
(L)

## 4. Discussion

ICC is insidious and easily invades the organs, tissues, and nerves around the liver [1, 23]. Limited understanding of its pathophysiology hinders early diagnosis and effective treatment in clinical practice, necessitating pathophysiological mechanism studies of its malignant biological behaviors [23].

Alterations in energy metabolism, particularly the aberrant activation of the aerobic glycolysis pathway in neoplastic cells, are critical indications of tumor malignancy. The high expression of NKILA has been found in ICC tissues and cells, which can promote cell proliferation to inhibit apoptosis and EMT, consistent with our previous findings [6]. Enrichment analysis showed that NKILA expression may be associated with autophagy and glycolytic pathways. We analyzed the Warburg effect and autophagy‐related indicators in clinical ICC samples, revealing upregulation of Glut1, LDH, and PKM2, alongside downregulation of LC3, implying a pronounced Warburg effect pathway and a suppressed autophagy pathway in ICC. These results were in line with published studies, where Pant et al. [24] reviewed the critical role of glycometabolic reprogramming in cholangiocarcinoma, and Khizar et al. [25] reviewed the role of autophagy and confirmed that regulating autophagy may be one of the pathways to inhibit EMT and progression of cholangiocarcinoma cells. Herein, in vitro experiment results demonstrated that NKILA silencing reduced the synthesis of Warburg effect‐related proteins and intermediate metabolites, while concurrently inhibiting autophagy‐associated immune escape. Conversely, the intervention of the Warburg effect inhibitor DCA counteracted the promoting impact of NKILA overexpression on these processes. Unlike previous studies that focused solely on the Warburg effect mediated by NKILA [26] or regarded NKILA merely as an autophagy‐related predictive molecule for diseases [27, 28], our findings, for the first time in ICC, establish a direct link between these two hallmark events through NKILA. This highlights our key discovery that NKILA not only directly regulates the Warburg effect, thus participating in the glycometabolic reprogramming, but also mediates autophagy‐associated immune escape.

The PD‐1/PD‐L1 signaling pathway induces immune escape and participates in promoting rapid tumor growth [29]. The activation of autophagy signaling pathways, including mTOR, can modulate the expression of PD‐L1. This study proved that NKILA suppressed autophagy‐related pathways by activating mTOR, decreased CD8^+^ T cell cytotoxicity, and consequently augmented PD‐L1 signaling that facilitated immune evasion in ICC cells and enhanced cancer progression. Gwangwa et al. [30] identified that Warburg effect and autophagy interact during tumorigenesis. Glycolysis leads to lactate production, which in turn generates an acidic and hypoxic microenvironment that promotes tumorigenesis, invasion, and metastasis, and the hypoxic microenvironment results in autophagy induction by increasing the production of ROS [30]. Accordingly, we also explored the correlation between the Warburg effect and autophagy. Our research findings indicated that the Warburg effect inhibitor DCA stimulated the autophagy pathway and counteracted the effects of NKILA overexpression on autophagy‐mediated immune evasion, which innovatively links the NKILA‐mediated Warburg effect to autophagy‐mediated immune evasion in the progression of ICC. While our findings demonstrate that DCA reversed the proimmune evasion effect of NKILA, which is consistent with existing studies highlighting the anticancer properties of DCA, the clinical application of DCA is limited by its side effects such as neurotoxicity [31]. Thus, the development of inhibitors targeting NKILA may be more conducive to the clinical treatment of ICC.

Furthermore, we measured MTX1 and TOMM40, which were highly expressed in ICC, as downstream mechanisms of NKILA. The lncRNAs can act as highly specific sensors of mRNAs, and this interaction leads to different posttranscriptional outcomes, including alterations in mRNA stability [32]. We observed that NKILA bound to MTX1 at specific sites and formed RNA double‐stranded bodies through RNA‐RNA interactions, which prevented MTX1 mRNA degradation and strengthened MTX1 stability. These findings represent the first demonstration of a regulatory interaction between NKILA and MTX1. In addition, through rescue experiments, we demonstrated that MTX1 overexpression reversed NKILA silencing‐suppressed tumor growth, cell viability, EMT, and Warburg effect, and NKILA silencing‐enhanced apoptosis and autophagy. These results suggested that the effect of NKILA on ICC progression may be mediated by its interaction with MTX1. MTX1 is a central member of mitochondrial transport [10]. MTX1 mediates autophagy, leading to sorafenib resistance in hepatocellular carcinoma cells or participating in aging [33]. This study demonstrated that MTX1 overexpression activated the mTOR pathway to inhibit the autophagy proteins LC3Ⅱ/LC3I and Beclin1, stimulate the Warburg effect, and upregulate glycolytic enzymes HK2, PKM2, and LDH, thus initiating the immune escape mechanism in ICC.

In the current study, we also demonstrate that MTX1 and TOMM40 were co‐locally expressed and bound, whereas NKILA silencing decreased mRNA and protein abundance of MTX1 and TOMM40 in vitro. Several studies have identified TOMM40 in Alzheimer’s disease [34–36]. It has also been reported that TOMM40 is involved in the process of mitophagy [37]. The link between TOMM40 and glycolysis has been indirectly demonstrated [38]. Here, we revealed that TOMM40 silencing reversed the effects of MTX1 overexpression on the malignant phenotype of ICC cells, the Warburg effect, and autophagy‐associated immune escape.

Taken together, our findings are the first to demonstrate that NKILA regulates the Warburg effect and autophagy‐associated immune escape by modulating the MTX1/TOMM40 axis in ICC, thereby providing novel insights into the pathogenesis of ICC. However, it still has limitations. First, preliminary studies suggest that NKILA has multiple potential cobinding partners, but this study only focuses on MTX1 and does not involve the interactions between NKILA and other molecules. Our understanding of its overall regulatory network still needs to be improved. Second, ICC exhibits high heterogeneity [39]. Although this study validated the findings with 10 pairs of clinical tissues and TCGA data, the results remain insufficient to cover the molecular characteristics of ICC across different pathological subtypes and clinical stages, so expanding the multicenter clinical sample sizes to verify the axis’s universality will be necessary in future research. Third, the study focused solely on mechanism elucidation and did not explore the potential of combination therapies targeting this axis in the context of clinically used ICC treatment regimens (e.g., chemotherapy, immunotherapy). Future studies should therefore evaluate the efficacy of relevant interventions through in vitro and in vivo models to facilitate clinical translation.

## 5. Conclusion

In conclusion, our findings reveal a novel molecular mechanism by which lncRNA‐NKILA stimulates the Warburg effect via the MTX1/TOMM40 pathway, innovatively linking lncRNA‐mediated glycometabolic reprogramming to autophagy‐dependent immune escape processes in ICC. These discoveries not only identify the NKILA‐MTX1/TOMM40 axis as a promising therapeutic target for ICC, but also lay a foundation for developing novel combination therapies that integrate metabolic modulation and immunotherapy to improve clinical outcomes.

**Nomenclature**
AFAP1: Actin filament associated protein 1
ALG6: Alpha‐1,3‐glucosyltransferase
ATP: Adenosine triphosphate levels
CCK‐8: Cell Counting Kit‐8
ChIP: Chromatin immunoprecipitation
Co‐IP: Coimmunoprecipitation
DCA: Dichloroacetate
DAPI: 4′,6‐diamidino‐2‐phenylindole
EdU: 5‐Ethynyl‐2′‐ deoxyuridine
EMT: Epithelial–mesenchymal transition
Glut1: Glucose transporter type 1
HK2: Hexokinase2
IHC: Immunohistochemistry
KD‐NC: Knockdown‐negative control
KD‐NKILA: Knockdown‐NKILA
KD‐TOMM40: Knockdown‐TOMM40
LC3: Light chain 3
LC3A/B: Light chain 3A/B
LDH: Lactate dehydrogenase
LDHA: Lactate dehydrogenase A
LncRNAs: Long noncoding RNAs
MCT1: Monocarboxylate transporter 1
mTOR: Mammalian target of rapamycin
MTX1: Metaxin 1
NC: Negative control
NKILA: Nuclear transcription factor NF‐κB interacting long noncoding RNA
OE‐MTX1: Overexpression‐MTX1
OE‐NKILA: Overexpression‐NKILA
P62: Sequestosome 1
p65: RelA
PBS: Phosphate buffered saline
PD‐L1: Programmed cell death 1 ligand 1
PK: Pyruvate kinase
PKM2: Pyruvate kinase isozyme typeM2
qRT‐PCR: Quantitative real‐time polymerase chain reaction
RPA: Ribonuclease protection assay
RPMI: Roswell Park Memorial Institute
SDS‐PAGE: Sodium dodecyl sulfate‐polyacrylamide gel electrophoresis
STRING: Search tool for recurring instances of neighboring genes
SUSD6: Sushi domain containing 6
TOMM40: Translocase of outer mitochondrial membrane 40
TUNEL: Terminal dexynucleotidyl transferase (TdT)‐mediated dUTP nick end labeling
UALCAN: The University of ALabama at Birmingham CANcer data analysis portal.

## Ethics Statement

Clinical or animal assays were approved by Ethics Committees of Shanghai Sixth People’s Hospital (2022‐YS‐285) or Zhejiang Baiyue Biotech Co., Ltd for Experimental Animals Welfare (ZJBYLA‐IACUC‐20231018). Clinical samples contained tumor tissues and adjacent tissues from 10 patients with intrahepatic cholangiocarcinoma. Animal experiments were conducted in accordance with the guidelines for the management and use of experimental animals.

## Conflicts of Interest

The authors declare no conflicts of interest.

## Author Contributions

**Meiying Zhu:** conceptualization (lead), writing – original draft (lead). **Hui Zhu:** formal analysis (lead), writing – review and editing (equal). **Zunqiang Zhou:** software (lead), writing – review and editing (equal). **Haiming Zheng:** methodology (lead), writing – review and editing (equal). **Zhixia Dong:** validation (lead), writing – original draft (supporting). **Wenhong Dong:** investigation (lead), writing – review and editing (equal). **Xinjian Wan:** supervision (lead), writing – review and editing (equal). Meiying Zhu and Hui Zhu contributed equally to this work.

## Funding

This work was sponsored by the Interdisciplinary Program of Shanghai Jiao Tong University (Grant YG2022QN086), Shanghai Science and Technology Innovation Action Plan (Grant 21JC1406603), National Natural Science Foundation of China (Grant 82374215), and the Interdisciplinary Program of Shanghai Jiao Tong University (Grant YG2023ZD20).

## Supporting Information

Additional supporting information can be found online in the Supporting Information section.

## Supporting information


**Supporting Information 1** Figure S1. (A–C) Protein quantification data corresponding to Figure 2D. (D–H) Protein quantification data corresponding to Figure 2E. (I–K) Protein quantification data corresponding to Figure 2G. (L,M) Protein quantification data corresponding to Figure 2H. (N) Protein quantification data corresponding to Figure 3E. (O). Protein quantification data corresponding to Figure 3Q. (P,Q) Protein quantification data corresponding to Figure 4B. (R–T) Protein quantification data corresponding to Figure 5A. Each experiment was repeated three times. Results are mean ± SD,  ^∗^ *p* < 0.05,  ^∗∗^ *p* < 0.01,  ^∗∗∗^ *p* < 0.001.

**Supporting Information 2** Figure S2. (A–E) Protein quantification data corresponding to Figure 5C. (F–H) Protein quantification data corresponding to Figure 5E. (I,J) Protein quantification data corresponding to Figure 5F. (K,L) Protein quantification data corresponding to Figure 6F. (M–Q) Protein quantification data corresponding to Figure 6G. (R–T) Protein quantification data corresponding to Figure 6K. (U,V) Protein quantification data corresponding to Figure 6L. Each experiment was repeated three times. Results are mean ± SD,  ^∗∗^ *p* < 0.01,  ^∗∗∗^ *p* < 0.001.

**Supporting Information 3** Figure S3. (A) Mouse models of ICC were constructed by implanting RBE cells. (B,C) Tumor volumes and weights of each group. (D,E) Flow cytometry was performed to analyze the effects of MTX1 overexpression and NKILA silencing on the percentage of CD8+ T cells. (F) NKILA mRNA expression was detected by qRT‐PCR. The internal parameter is β‐actin. (G–I) MTX1 and TOMM40 protein levels were detected by western blot. (J–L) Warburg‐related indices were determined by IHC (scale: 100 μm). Each experiment was repeated three times.  ^∗∗^ *p* < 0.01,  ^∗∗∗^ *p* < 0.001.

## Data Availability

The data that support the findings of this study are available from the corresponding author upon reasonable request.
