# Supplementary figures and images for: lncRNA NKILA Promotes Warburg Effect and Immune Escape in Intrahepatic Cholangiocarcinoma by Regulating the MTX1/TOMM40 Axis
Source: Mediators Inflamm. 2025 Dec 22;2025:7712817. doi: 10.1155/mi/7712817 (PMC12767443; doi:10.1155/mi/7712817)

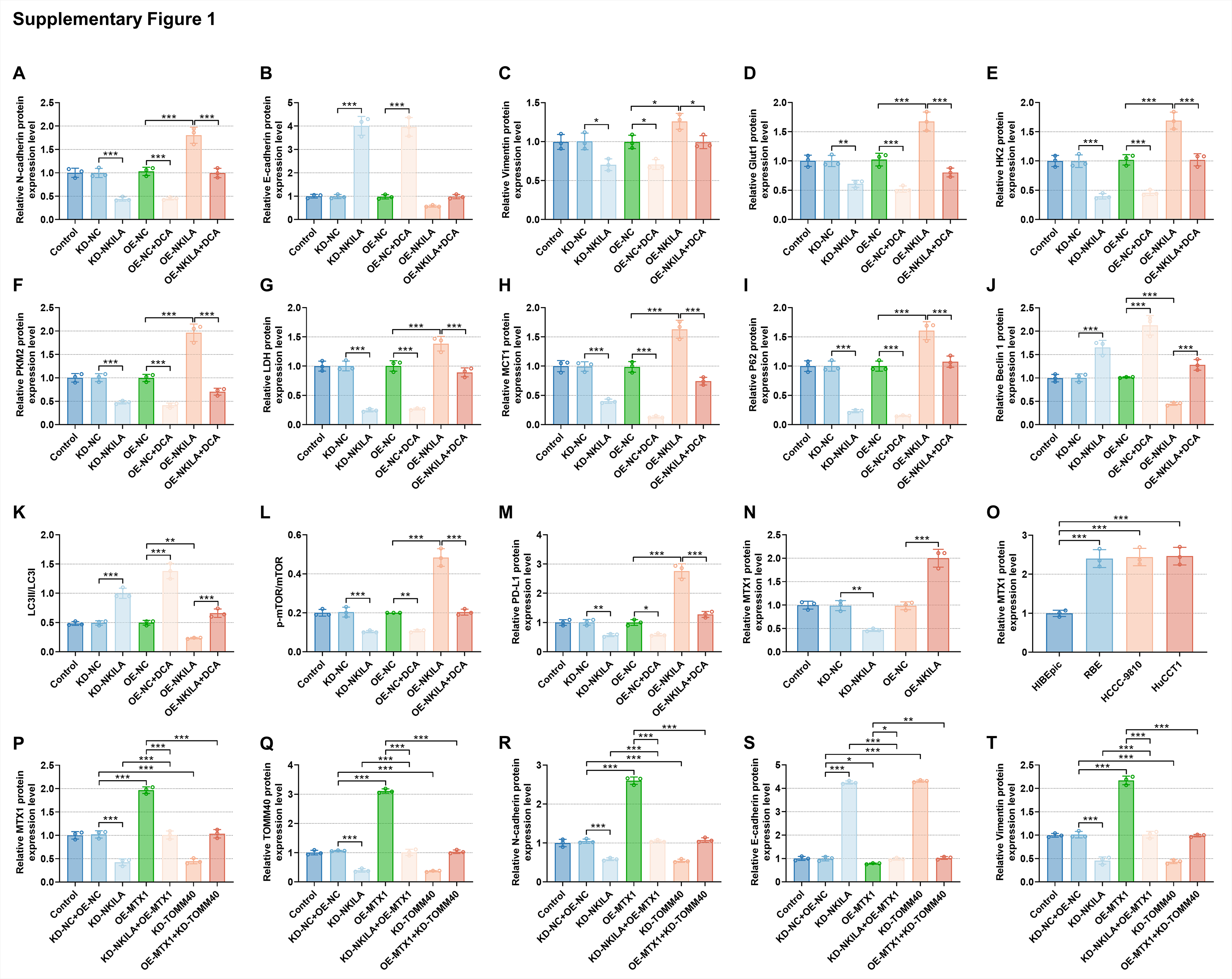

Supplement: Supplementary file 1 — Supporting Information 1 Figure S1. (A–C) Protein quantification data corresponding to Figure 2D. (D–H) Protein quantification data corresponding to Figure 2E. (I–K) Protein quantification data corresponding to Figure 2G. (L,M) Protein quantification data corresponding to Figure 2H. (N) Protein quantification data corresponding to Figure 3E. (O). Protein quantification data corresponding to Figure 3Q. (P,Q) Protein quantification data corresponding to Figure 4B. (R–T) Protein quantification data corresponding to Figure 5A. Each experiment was repeated three times. Results are mean ± SD, ∗ p < 0.05, ∗∗ p < 0.01, ∗∗∗ p < 0.001. [file MI-2025-7712817-s001.tif]

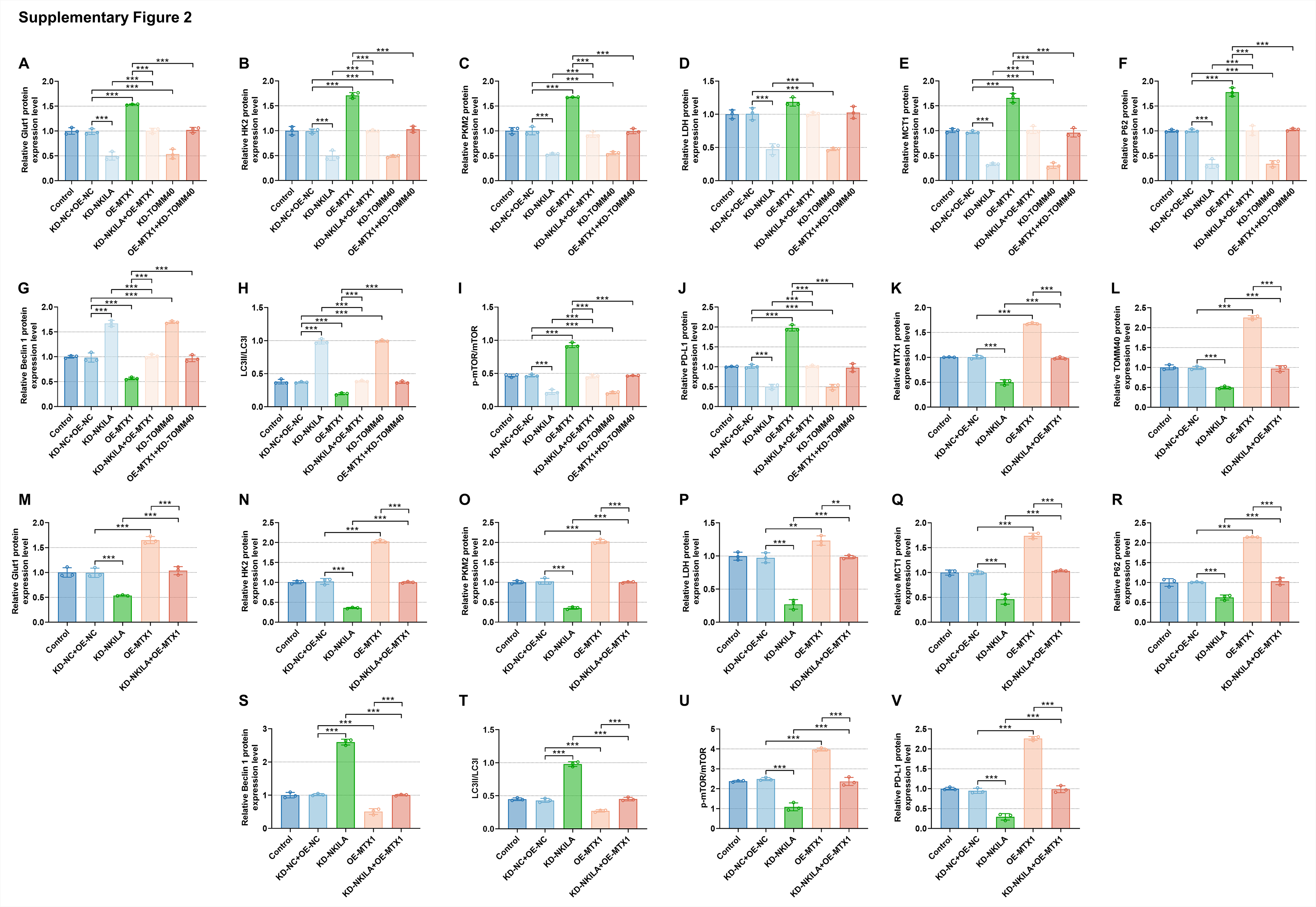

Supplement: Supplementary file 2 — Supporting Information 2 Figure S2. (A–E) Protein quantification data corresponding to Figure 5C. (F–H) Protein quantification data corresponding to Figure 5E. (I,J) Protein quantification data corresponding to Figure 5F. (K,L) Protein quantification data corresponding to Figure 6F. (M–Q) Protein quantification data corresponding to Figure 6G. (R–T) Protein quantification data corresponding to Figure 6K. (U,V) Protein quantification data corresponding to Figure 6L. Each experiment was repeated three times. Results are mean ± SD, ∗∗ p < 0.01, ∗∗∗ p < 0.001. [file MI-2025-7712817-s003.tif]

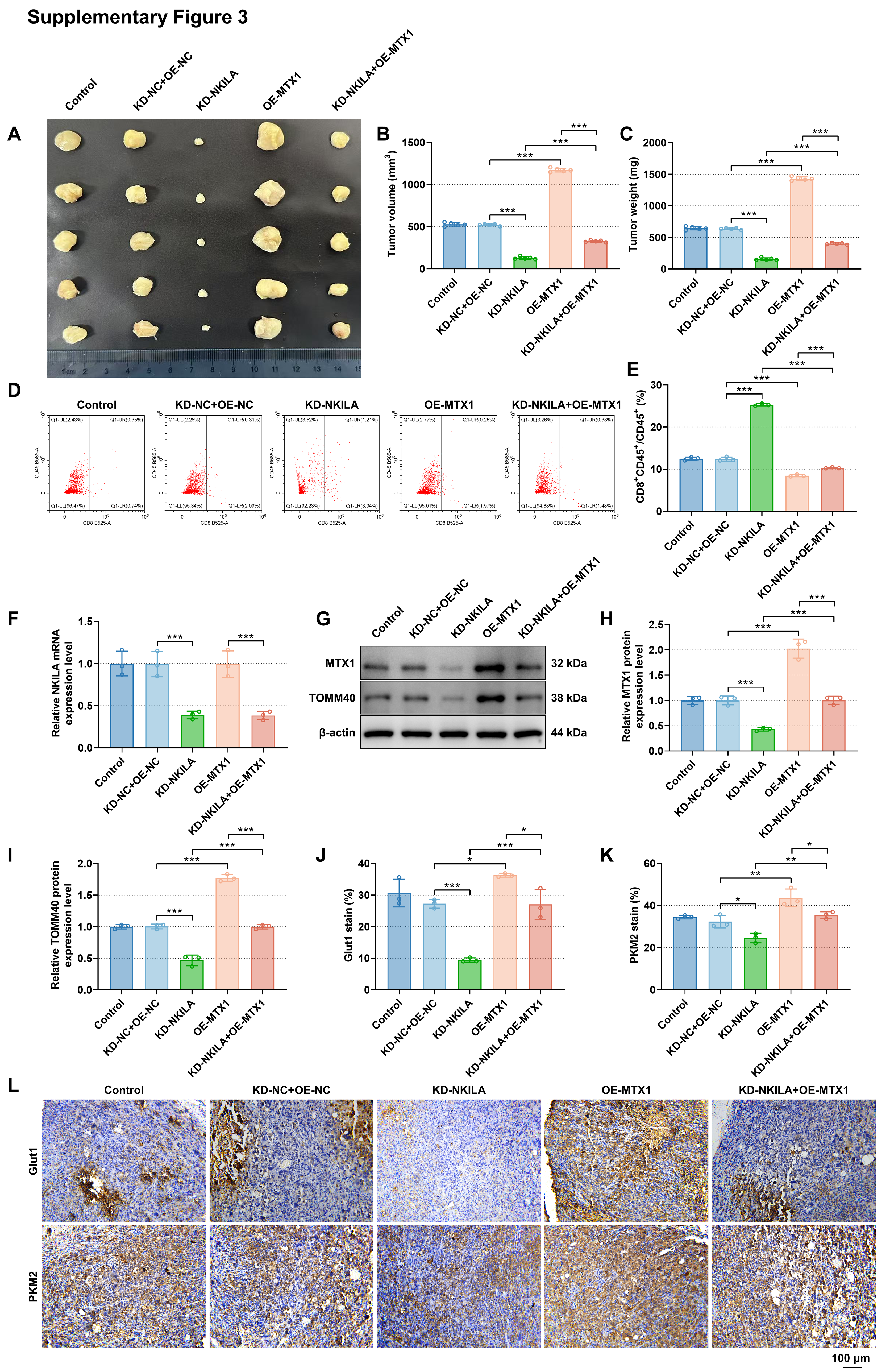

Supplement: Supplementary file 3 — Supporting Information 3 Figure S3. (A) Mouse models of ICC were constructed by implanting RBE cells. (B,C) Tumor volumes and weights of each group. (D,E) Flow cytometry was performed to analyze the effects of MTX1 overexpression and NKILA silencing on the percentage of CD8+ T cells. (F) NKILA mRNA expression was detected by qRT‐PCR. The internal parameter is β‐actin. (G–I) MTX1 and TOMM40 protein levels were detected by western blot. (J–L) Warburg‐related indices were determined by IHC (scale: 100 μm). Each experiment was repeated three times. ∗∗ p < 0.01, ∗∗∗ p < 0.001. [file MI-2025-7712817-s002.tif]
